# The Acute Toxicity of Mineral Fibres: A Systematic In Vitro Study Using Different THP-1 Macrophage Phenotypes

**DOI:** 10.3390/ijms23052840

**Published:** 2022-03-04

**Authors:** Serena Mirata, Vanessa Almonti, Dario Di Giuseppe, Laura Fornasini, Simona Raneri, Stefania Vernazza, Danilo Bersani, Alessandro F. Gualtieri, Anna Maria Bassi, Sonia Scarfì

**Affiliations:** 1 Department Earth, Environment and Life Sciences, University of Genova, 16132 Genova, Italy; serenamira94@gmail.com; 2 Inter-University Center for the Promotion of the 3Rs Principles in Teaching & Research (Centro 3R), 56122 Pisa, Italy; vanessaalmonti@gmail.com (V.A.); stefania.vernazza@yahoo.it (S.V.); anna.maria.bassi@unige.it (A.M.B.); 3 Department Experimental Medicine, University of Genova, 16132 Genova, Italy; 4 Department of Chemical and Geological Sciences, University of Modena and Reggio Emilia, Via G. Campi 103, 41125 Modena, Italy; dario.digiuseppe@unimore.it (D.D.G.); alessandro.gualtieri@unimore.it (A.F.G.); 5 ICCOM-CNR—Institute of Chemistry of OrganoMetallic Compounds, National Research Council, Via G. Moruzzi 1, 56124 Pisa, Italy; laura.fornasini@pi.iccom.cnr.it (L.F.); simona.raneri@pi.iccom.cnr.it (S.R.); 6 Department of Mathematical, Physical and Computer Sciences, University of Parma, Parco Area delle Scienze 7/A, 43124 Parma, Italy; danilo.bersani@unipr.it

**Keywords:** mineral fibres, asbestos, carcinogenicity, apoptosis, DNA double-strand breaks

## Abstract

Alveolar macrophages are the first line of defence against detrimental inhaled stimuli. To date, no comparative data have been obtained on the inflammatory response induced by different carcinogenic mineral fibres in the three main macrophage phenotypes: M0 (non-activated), M1 (pro-inflammatory) and M2 (alternatively activated). To gain new insights into the different toxicity mechanisms of carcinogenic mineral fibres, the acute effects of fibrous erionite, crocidolite and chrysotile in the three phenotypes obtained by THP-1 monocyte differentiation were investigated. The three mineral fibres apparently act by different toxicity mechanisms. Crocidolite seems to exert its toxic effects mostly as a result of its biodurability, ROS and cytokine production and DNA damage. Chrysotile, due to its low biodurability, displays toxic effects related to the release of toxic metals and the production of ROS and cytokines. Other mechanisms are involved in explaining the toxicity of biodurable fibrous erionite, which induces lower ROS and toxic metal release but exhibits a cation-exchange capacity able to alter the intracellular homeostasis of important cations. Concerning the differences among the three macrophage phenotypes, similar behaviour in the production of pro-inflammatory mediators was observed. The M2 phenotype, although known as a cell type recruited to mitigate the inflammatory state, in the case of asbestos fibres and erionite, serves to support the process by supplying pro-inflammatory mediators.

## 1. Introduction

Exposure to mineral fibres represents a serious occupational and environmental hazard causing the emergence of fibrotic pulmonary diseases, pneumoconiosis and various types of cancer in exposed subjects [1]. One of the consequences is a high number of casualties and elevated costs for the healthcare systems of both developed and undeveloped countries. Thus, understanding the mechanisms of toxicity and carcinogenicity of inhalable mineral fibres constitutes a fundamental step towards the quantitative classification of the toxicity/carcinogenicity of mineral fibres for preventive medicine and the development of effective treatments for both at-risk workers and the general population.

Macrophages in the lung constitute the first and foremost line of defence against detrimental inhaled stimuli. They comprise a population of myeloid cells that belong to the innate immune system and play a fundamental role in both triggering and maintaining the inflammatory response that develops in the tissues as a consequence of harmful stimuli. In fact, cell damage is a necessary step in triggering the inflammatory response of immune cells. These actors are differentiated mainly by the blood and bone marrow precursors that accumulate in the tissue due to chemokines released from injured cells [2]. Once recruited to the site of inflammation in the specific tissue, macrophages have the capacity to adapt their phenotype in response to different local stimuli, displaying a high degree of plasticity. In the lung, for instance, a limited number of macrophage sentinels, i.e., resident alveolar macrophages [3], rapidly localise to the site of infection or injury and initiate the inflammatory process. Inflammation prompts the release of chemokines with the function of recruiting circulating monocytes and causing their extravasation and maturation in the tissue to non-activated (M0) macrophages. Subsequently, the newly differentiated M0 macrophages are further polarised by local stimuli in the tissue to phenotypes defined as classically activated type 1 (M1) and alternatively activated type (M2) macrophages [4]. Classically activated M1 macrophages are found at the site of damage shortly after injury or infection, with their phenotype developing in the presence of bacterial endotoxin (LPS) and IFN-γ or TNF-α. They release pro-inflammatory cytokines (i.e., TNF-α, IL-1β, IL-6, IL-12 and IL-15) and promote the production of cytotoxic reactive oxygen species (ROS), proteolytic enzymes and bioactive lipids. Conversely, M2 macrophages, which can be polarised by IL-4 and/or IL-13 stimuli, may appear later at the site of inflammation and counteract the pro-inflammatory activity of the M1 phenotype by secreting anti-inflammatory mediators (i.e., IL-10, CCL18 and CCL22) and tissue-repair-promoting factors, such as TGF-β, VEGF and FGF [5]. However, while M1 and M2 cells were initially assumed to be phenotypically and functionally distinct subpopulations with opposite roles in the inflammatory response, it is now recognised that macrophages exist as several subpopulations with different levels of M1/M2 markers and activities [6,7].

A very useful experimental model that can provide insights into the modulation of monocyte and macrophage functions and behaviour is the human monocytic cell line THP-1. These cells can be easily differentiated into the M0, M1 and M2 phenotypes in culture; they can then be co-cultured with a plethora of different cell types to mimic tissue-specific interactions and be challenged by a large variety of stimuli, such as infectious agents, asbestos and pollutants [8]. This in vitro human cell model has previously been used in several studies with the aim of understanding the mechanisms of acute toxicity and the inflammatory response of macrophages towards toxic and carcinogenic mineral fibres (see the recent paper by our group, [9]). All of the known previous studies always focused on one type of THP-1-derived phenotype, namely, M0 macrophages, which were usually challenged by the most important commercial asbestos fibres: chrysotile [10,11,12,13,14,15] and crocidolite [16,17,18,19,20,21,22]. To date, no comparative data have been obtained from the concomitant analysis of the inflammatory response induced by different mineral fibres in the three macrophage phenotypes, namely, non-activated M0, pro-inflammatory M1 and alternatively activated M2 cells. Indeed, all three phenotypes can be present at the site of injury and contribute to the outcomes of the inflammatory process. Thus, to fill this gap and gain new insights into the different mechanisms of the toxicity of mineral fibres, the three M0, M1 and M2 macrophage phenotypes, obtained from THP-1 cell differentiation, were used to study the acute effects of three types of carcinogenic mineral fibres: fibrous erionite, crocidolite and chrysotile.

Asbestos is a generic term used to identify six mineral fibres of commercial and economic importance: five amphibole species (i.e., amphibole asbestos) and chrysotile (a member of serpentine asbestos) [23,24,25]. The family of amphibole asbestos includes actinolite asbestos, amosite, anthophyllite asbestos, crocidolite and tremolite asbestos. Amphiboles are double-chain silicates with the general formula A_0-1_B_2_C_5_T_8_O_22_W_2_, where A = Na^+^, K^+^, Ca^2+^, Li^+^ (cation sites with 8- to 12-fold coordination); B = Na^+^, Li^+^, Ca^2+^, Mn^2+^, Fe^2+^, Mg^2+^ (less regular octahedral or 8-fold coordinated cation sites); C = Mg^2+^, Fe^2+^, Mn^2+^, Al^3+^, Fe^3+^, Ti^3+^, Ti^4+^ (fairly regular octahedral cation sites); and T = Si^4+^, Al^3+^ (tetrahedral sites of the silicate chain) [24,26]. The growth of the crystals occurs along the c-axis in amphibole asbestos and is responsible for their fibrous nature. The most relevant mineral fibre of the amphibole asbestos group is crocidolite. Crocidolite, also called blue asbestos, is the fibrous/asbestiform variety of riebeckite, i.e., a sodic amphibole with the ideal formula Na_2_(Fe^2+^_3_Fe^3+^_2_)[Si_8_O_22_](OH)_2_. Detailed and up-to-date information on the classification, crystal chemistry and structural characteristics of amphibole asbestos is provided in the literature [26].

Chrysotile is the most common asbestos mineral and belongs to the serpentine group [23,24]. Serpentine minerals are layer silicates composed of Si-centred tetrahedral (T) sheets in a pseudo-hexagonal network joined to Mg-centred octahedral (O) sheets in units with a 1:1 (TO) ratio. Since the TO unit is polar and a misfit exists between the smaller T sheet and the larger O sheet, a differential strain occurs between the two sheets [26]. The strain is released by rolling the TO layer around a preferred axis, leading to the formation of the tubular structure typical of chrysotile fibres [24,26]. The general chemical formula of chrysotile is Mg_3_(OH)_4_Si_2_O_5_. The most common substitution occurs between Fe^2+^ and Mg^2+^ in the octahedral site [26]. Moreover, Al^3+^ and Fe^3+^ can replace both Si^4+^ and Mg^2+^ in the tetrahedral and octahedral sites. Among the different forms of asbestos, chrysotile is the most extensively used worldwide in the manufacturing of brake and clutch linings, insulation systems, cement boards and roofing tiles, to cite a few [27].

Erionite is a natural zeolite belonging to the so-called ABC-6 family whose structure is composed of columns of cancrinite cages connected by a double six-membered ring of tetrahedra, forming hexagonal prisms [26,28]. The ideal formula of erionite is K_2_(Na, Ca_0.5_)_7_[Al_9_Si_27_O_72_]·28H_2_O. Erionite is characterised by a large chemical variability, with three different species identified according to the most abundant extra-framework cation: erionite-Na, erionite-K and erionite-Ca. 

Asbestos and fibrous erionite are known toxic and pathogenic agents and are included in Group 1, carcinogenic to humans, by the International Agency for Research on Cancer (IARC) [25]. 

This work is part of a long-term Italian Research Project of National Interest (PRIN) that has been in progress since 2017, the aim of which is to understand the biochemical mechanisms leading to adverse effects in vivo associated with exposure to mineral fibres. For this project, we chose three mineral fibres as reference samples, namely: Union for International Cancer Control (UICC) standard crocidolite, chrysotile from Balangero (manufacture city, Turin, Italy) and erionite from Jersey (Nevada, USA). The three mineral fibres are representative of the classes of amphibole asbestos, serpentine asbestos and fibrous erionite, respectively. Supplementary Figure S1 shows a selection of electron microscope images of the investigated fibres. A detailed description of these fibres, as well as their size distribution when dispersed in the cell culture medium, has been reported previously [9]. Summary statistics of the lengths and widths of the fibres are shown in Table 1.

The goal of this study was to investigate the different possible toxicity mechanisms exerted by the three mineral fibres in the first 24 h after challenge and look into early mutagenic signs (i.e., DNA damage) by performing a comparative study in an in vitro THP-1 cellular model of the three macrophage phenotypes, M0, M1 and M2. Their cytotoxic action was studied by measuring the level of cell death by either cell lysis or apoptosis. Subsequently, the intracellular oxidative state, intracellular toxic metal release, the presence of DNA damage and the inflammatory response were studied in the three cell phenotypes, as these are recognised factors that occur early during the inflammatory response and contribute to the onset of chronic diseases and malignancies in the long term. Our results aim to fill the gap in the understanding of the fine biochemical interactions of different types of carcinogenic fibres inside cells, hopefully helping to upgrade existing models of the toxicity/carcinogenicity of mineral fibres.

## 2. Results and Discussion

### 2.1. Cell–Fibre Interaction Imaging and Fibre Surface Characterisation

**Figure 1:**
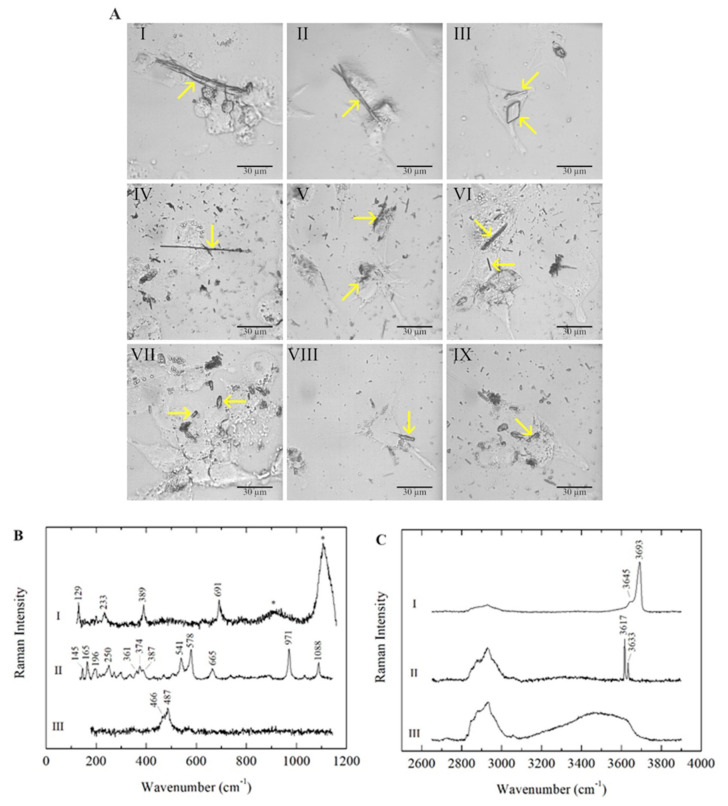
(**A**) Microscope images of M0, M1 and M2 macrophages in the presence of mineral fibres chrysotile, crocidolite and erionite. Panels I, II and III: chrysotile-treated M0, M1 and M2 macrophages, respectively. Panels IV, V and VI: crocidolite-treated M0, M1 and M2 macrophages, respectively. Panels VII, VIII and IX: erionite-treated M0, M1 and M2 macrophages, respectively. Typical morphologies of the fibres are highlighted with yellow arrows. The scale bar represents 30 μm. (**B**) Raman spectra of the mineral fibres chrysotile (I), crocidolite (II) and erionite (III) in M0 macrophages, acquired in the low-wavenumber spectral range. Photoluminescence bands of Cr^3+^ emissions are marked with asterisks (*). (**C**) Raman spectra of the mineral fibres chrysotile (I), crocidolite (II) and erionite (III) in M0 macrophages, acquired in the high-wavenumber spectral range. The Raman bands between ~2800 and 3100 cm*^−^*^1^ are attributed to CH stretching modes due to the cellular signals.

In all three types of polarised macrophages (M0, M1 and M2), the mineral fibres were detected inside the cells and identified by micro-Raman analyses after 24 h of incubation. As expected, chrysotile (CHR), crocidolite (CRO) and erionite (ERI) fibres showed different morphologies (Figure 1A and Figure S1). 

In all three investigated mineral fibres, impurities consisting of non-fibrous micrometric crystals were detected inside the cells along with fibrous phases, as previously reported in the literature (Table 1). In CHR-treated macrophages, bundles of curvilinear chrysotile fibres with splayed/frayed terminations and thinner and shorter single fibres were observed (Figure 1A, panels I, II and III); minor contributions of other minor fibrous and lamellar phases (balangeroite and antigorite, respectively) were also detected in addition to the non-fibrous content. CRO fibres showed a thin and rigid morphology with variable lengths (Figure 1A, panels IV, V and VI). ERI fibres were typically shorter than the other mineral fibres, exhibiting a more uniform size distribution (Figure 1A, panels VII, VIII and IX). The mineral fibres inside the three types of differentiated phagocytic cells were identified by their characteristic Raman spectra, acquired in both the low- and high-wavenumber spectral ranges, and the positions of the main peaks were highlighted (Figure 1B,C). For serpentine minerals, the lattice and internal vibrational modes were similar in different polymorphs, demonstrating that OH stretching signals are distinctive characteristics for the identification of the mineralogical phase in CHR-treated cells [29,30]. In addition to CHR (curve I in Figure 1B,C), the lamellae of antigorite and balangeroite fibres were detected as minor contributions (Table 1) [31]. Additionally, micrometric crystals consisting of impurities were observed in CHR-treated macrophages, highlighting the presence of iron compounds (Table 1) [32]. Furthermore, in the low range, it was possible to detect the photoluminescence bands of Cr^3+^ emissions at ~671 and 680 nm due to the presence of chromium as a trace element in the CHR fibres (marked with asterisks in curve I in Figure 1B) [33]. In CRO-treated macrophages, CRO was identified by its peculiar Raman spectrum (curve II in Figure 1B,C) [34,35], and minor phases—including iron-bearing micro-crystals—were observed (Table 1) [36,37]. Erionite was the main fibrous mineral phase detected in ERI-treated macrophages, with minor tabular clinoptilolite [38]. Here, iron-bearing impurities—as identified in SEM and TEM analyses in previous studies [28]—were not observed through micro-Raman analyses, as they are generally smaller than the minimum detectable size, considering the spatial resolution of the spectrometer. It is noteworthy that for all three types of polarised macrophages, the Raman signals of cells were always found along with those of the analysed mineral fibres, and no differences in the signals were detected for each mineral fibre in the three types of differentiated macrophages. Cellular signals were especially clear from their CH stretching bands in the spectral range ~2800–3100 cm^−1^ (Figure 1C).

**Table 1:** General information on the mineral fibres investigated in this study. Impurities detected in the present work and in [28,32,36,37]. Fibre lengths and widths from Reference [9].

Mineral Fibre	Impurities	Fibre Length (µm)	Fibre Width (µm)
Chrysotile (Balangero, Turin, Italy)	Antigorite, balangeroite, calcite, clinochlore, diopside, dolomite, magnetite, microcline, plagioclase, talc, mackinawite, hematite, ilmenite, lepidocrocite, Fe-Ni sulphide, Fe-Mg carbonate	* Min: 4.02 Mean: 34.7 ** Max: 188	Min: 0.18 Mean: 0.59 Max: 1.17
Crocidolite (UICC)	Hematite, magnetite, quartz, talc, lizardite, calcite, siderite, minnesotaite	Min: 2.52 Mean: 16.1 Max: 131	Min: 0.23 Mean: 0.64 Max: 1.98
Erionite (Jersey, Nevada, USA)	Clinoptilolite, iron-rich nanoparticles, iron oxides/hydroxides, nontronite	Min: 3.23 Mean: 9.39 Max: 55.0	Min: 0.25 Mean: 0.55 Max: 6.70

* Minimum; ** Maximum.

### 2.2. Acute Toxicity of Mineral Fibres

**Figure 2:**
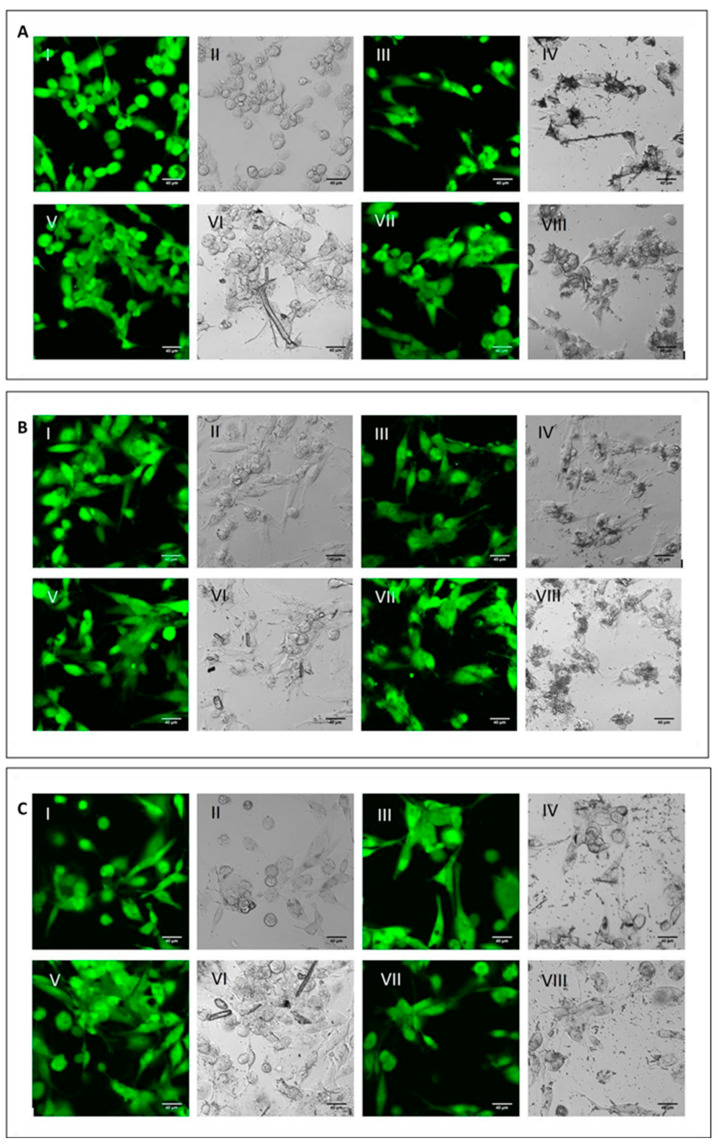
Qualitative evaluation of short-term cytotoxicity. (**A**) Visualisation by confocal microscopy analysis (2.5 × digital zoom) in fluorescence mode (panels I, III, V and VII) and in phase contrast (panels II, IV, VI and VIII) of M0 macrophages following 4 h of incubation in the presence or absence of mineral fibres at 50 µg/mL and staining with calcein-AM. Panels I-II: control; panels III-IV: CRO; panel V-VI: CHR; panels VII-VIII: ERI. The white bar represents 40 µm. (**B**) Visualisation of pro-inflammatory M1 macrophages in the same conditions as cells in panel (**A**). (**C**) Visualisation of alternatively activated M2 macrophages in the same conditions as cells in panel (**A**).

To analyse the cell viability and the phagocytic ability of M0, M1 and M2 macrophages, the differentiated cells were incubated with the three mineral fibres. After 4 h of incubation, we used calcein-AM staining—a green-fluorescent dye used to label live cells—to determine if the phagocytic cells had engaged and internalised the particles and if this had already affected cell viability (Figure 2). 

For all three macrophage types, M0 (Figure 2A), M1 (Figure 2B) and M2 (Figure 2C), there were no differences in fluorescence positivity between control cells (panels AI-II, BI-II and CI-II for M0, M1 and M2 macrophages, respectively) and fibre-treated cells (panels III-IV CRO, panels V-VI CHR and panels VII-VIII ERI, respectively). In fact, in all experimental conditions, cells showed intense green cytoplasmic fluorescence, meaning that after 4 h of treatment with the fibres, no signs of cytotoxicity were visible yet. Nonetheless, in all types of macrophages, it was possible to observe the presence of the different types of mineral fibres inside the cytoplasm of the cells when the size of the fibres was short enough to allow their phagocytosis. From a qualitative point of view, M2 macrophages showed a higher number of mineral fibres dispersed at the bottom of the well, especially for CRO (panel C-IV) and ERI (panel C-VIII), compared to M0 and M1 macrophages (panels A and B), which had already captured many fibres or were in direct contact with them. The observed phenomenon may indicate that M2 macrophages are less able to engulf the mineral fibres compared to the other two phenotypes. This is plausible since this type of macrophage polarisation should have a more anti-inflammatory role [7]. 

**Figure 3:**
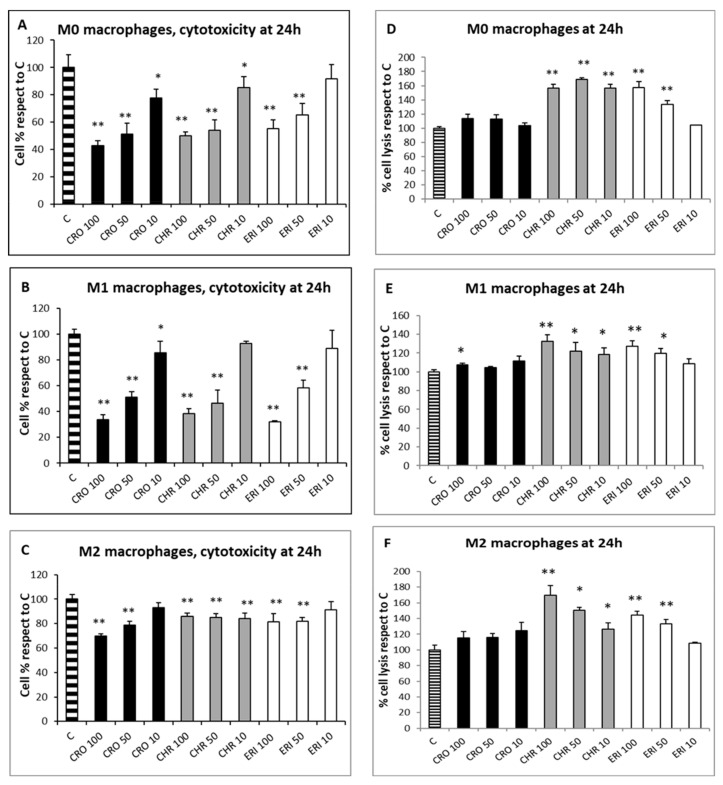
Cell toxicity evaluation and cell lysis assessment after exposure to mineral fibres. (**A**) M0 macrophage cytotoxicity evaluation by the MTT test at 24 h in the presence of 100, 50 and 10 μg/mL of the mineral fibres CRO (black bars), CHR (grey bars) and ERI (white bars). Results are expressed as cell percentages relative to control cells (striped bar) and are the mean ± SD of three independent experiments performed in quadruplicate. Asterisks indicate the significance in a paired Tukey test (ANOVA, *p* < 0.0001; Tukey vs. C: * *p* < 0.05, ** *p* < 0.005, respectively). (**B**) Pro-inflammatory M1 macrophage cytotoxicity evaluation in the same conditions as (**A**). ANOVA, *p* < 0.000005; Tukey vs. C: * *p* < 0.05, ** *p* < 0.005, respectively. (**C**) Alternatively activated M2 macrophage cytotoxicity evaluation in the same conditions as (**A**). ANOVA, *p* < 0.000001; Tukey vs. C: ** *p* < 0.005. (**D**) Cell lysis assessment measured by quantification of LDH release in the cell medium at 24 h in M0 macrophages after incubation with 100, 50 and 10 μg/mL of the mineral fibres CRO (black bars), CHR (grey bars) and ERI (white bars). Results are expressed as percentage of cell lysis relative to control cells (striped bar) and are the mean ± SD of three experiments performed in quadruplicate. Asterisks indicate significance in paired Tukey test (ANOVA, *p* < 0.0005; Tukey vs. C: ** *p* < 0.005). (**E**) Cell lysis assessment measured in the same conditions as (**D**) in pro-inflammatory M1 macrophages. ANOVA, *p* < 0.01; Tukey vs. C: * *p* < 0.05, ** *p* < 0.005, respectively. (**F**) Cell lysis assessment measured in the same conditions as (**D**) in alternatively activated M2 macrophages. ANOVA, *p* < 0.01; Tukey vs. C: * *p* < 0.05, ** *p* < 0.005, respectively.

Since the qualitative assessment of macrophage viability by calcein staining showed no significant signs of toxicity after 4 h of fibre treatment, the MTT test was used to evaluate the cytotoxic effects of CRO, CHR and ERI (Figure 3, panels A–C, black, grey and white bars, respectively) in macrophages after 24 h. This assay measures mitochondrial enzyme activity by quantifying resultant alterations of energy metabolism and, ultimately, cell death. The viability index showed that there was a dose-dependent toxic effect resulting in a significant cell death rate in both M0 and M1 macrophages after exposure to all three fibres, while CRO exhibited a similar behaviour only in M2 cells. In M0 macrophages (Figure 3A, black bars), CRO caused a dose-dependent cell death rate of 57%, 49% and 22% at 100, 50 and 10 µg/mL, respectively, compared to control cells. Similarly, CHR (grey bars) induced cell death rates of 49%, 46% and 14% at the same fibre concentrations compared to control cells. In M0 macrophages, ERI (white bars) showed slightly lower toxicity compared to asbestos fibres, with significant death rates (45% and 35%) only at the two highest fibre concentrations (100 and 50 µg/mL, respectively) compared to control cells.

Exposure to the three mineral fibres also induced a relevant cytotoxic effect in M1 macrophages (Figure 3B). The dose-dependent cell death rate after exposure to CRO was higher in M1 cells than in M0 cells (66%, 49% and 14% at 100, 50 and 10 µg/mL, respectively, compared to control cells). CHR at 100 and 50 µg/mL was also more cytotoxic in M1 than in M0, with cell death percentages ranging from 61% to 53%, respectively, compared to control cells. Regarding ERI, a strong cytotoxic effect was observed at 100 and 50 µg/mL (68% and 41%, respectively, compared to control cells).

M2 macrophages (Figure 3C) demonstrated a dose-dependent cytotoxic effect during exposure to CRO, although the cell death rates were lower than those found in M0 and M1 cells, with percentages ranging from 30% to 21% at 100 and 50 µg/mL, respectively, compared to control cells. CHR showed minor but significant cytotoxicity; cell death values were similar at all concentrations and around 14–15% compared to control cells (*p* < 0.005 vs. C for all). Finally, ERI slightly induced cell death in M2 cells only at 100 and 50 µg/mL, with a 19% reduction in viability at both concentrations compared to control cells (*p* < 0.05 vs. C for both). Overall, CRO and CHR showed a higher acute toxicity rate, strongly affecting energy metabolism and causing a significant cell death rate, than ERI in M0, M1 and M2 macrophages. In particular, for CRO treatment, our results are in line with a previous work [22], where a similarly high THP-1 macrophage cytotoxicity rate was observed in the first 24 h of exposure. Furthermore, lower fibre-induced cytotoxicity was observed in M2 macrophages compared to the other two phenotypes; this finding can be ascribed to their anti-inflammatory role and consequent lower phagocytic activity, leading to a reduced cell death rate due to frustrated phagocytosis.

To assess the nature of macrophage cell death, two analyses were performed: the lactate dehydrogenase (LDH) assay, which can be considered a cell death marker since the leakage of this cytosolic enzyme in the cell medium occurs following plasma membrane disturbance, and the annexin/propidium iodide positivity assay, measured by confocal microscopy to establish the level of cellular apoptosis. The LDH assay was performed on the three types of differentiated macrophages in contact with the fibres at three concentrations (100, 50 and 10 µg/mL) for 24 h to quantify cell death caused by plasma membrane damage (Figure 3D–F). The assay showed that, for all of the polarised macrophages, the rates of cellular damage observed after exposure to CRO were comparable to the control (Figure 3D–F black bars). Conversely, all types of differentiated macrophages showed a certain degree of plasma membrane cell damage during treatment at all concentrations of CHR (grey bars) and at the two highest concentrations of ERI (100 and 50 µg/mL, white bars). In particular, in M0 cells (Figure 3D), the degree of cellular damage induced by CHR was not dose-dependent, and the relative values of LDH release were 1.56-, 1.68- and 1.56-fold higher than control cells at 100, 50 and 10 µg/mL, respectively, while for ERI, significant dose-dependent plasma membrane cell damage was observed, with values 1.58- and 1.33-fold higher than control cells at the two highest fibre concentrations. The trend was similar in M1 macrophages (Figure 3E), although levels of cell membrane damage were observed to be lower compared to M0 cells. In particular, for CHR treatment, the increase in necrotic events compared to control cells was 1.32-, 1.22- and 1.18-fold at 100, 50 and 10 µg/mL, respectively, while for ERI, it amounted to 1.27- and 1.19-fold compared to control cells at 100 and 50 µg/mL.

The same trend was also evident in M2 cells (Figure 3F), since all tested concentrations of CHR induced an increase in cell lysis in the culture (1.69-, 1.50- and 1.26-fold increase at 100, 50 and 10 µg/mL, respectively), as was also observed with 100 and 50 µg/mL of ERI (1.44- and 1.33-fold for 100 and 50 µg/mL, respectively).

**Figure 4:**
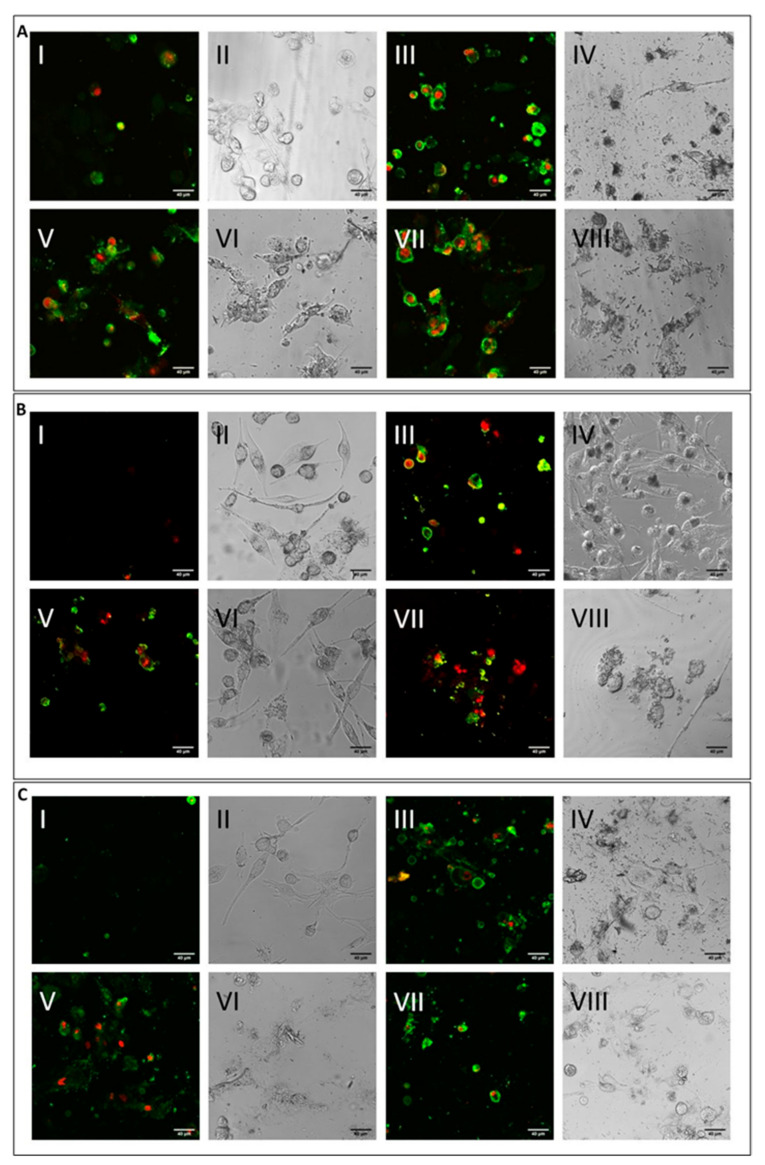
Cell apoptosis assessment by confocal microscopy analysis. (**A**) Visualisation by confocal microscopy (2.5 × digital zoom) in fluorescence mode (panels I, III, V and VII) and in phase contrast (panels II, IV, VI and VIII) of M0 macrophages following 24 h of incubation in the presence or absence of mineral fibres at 50 µg/mL and staining of annexin-positive (green) and/or propidium iodide–positive (red) cells. Panels I-II: control; panels III-IV: CRO; panels V-VI: CHR; panels VII-VIII: ERI. The white bar represents 40 µm. (**B**) Visualisation of pro-inflammatory M1 macrophages in the same conditions as cells in panel (**A**). (**C**) Visualisation of alternatively activated M2 macrophages in the same conditions as cells in panel (**A**).

**Figure 5:**
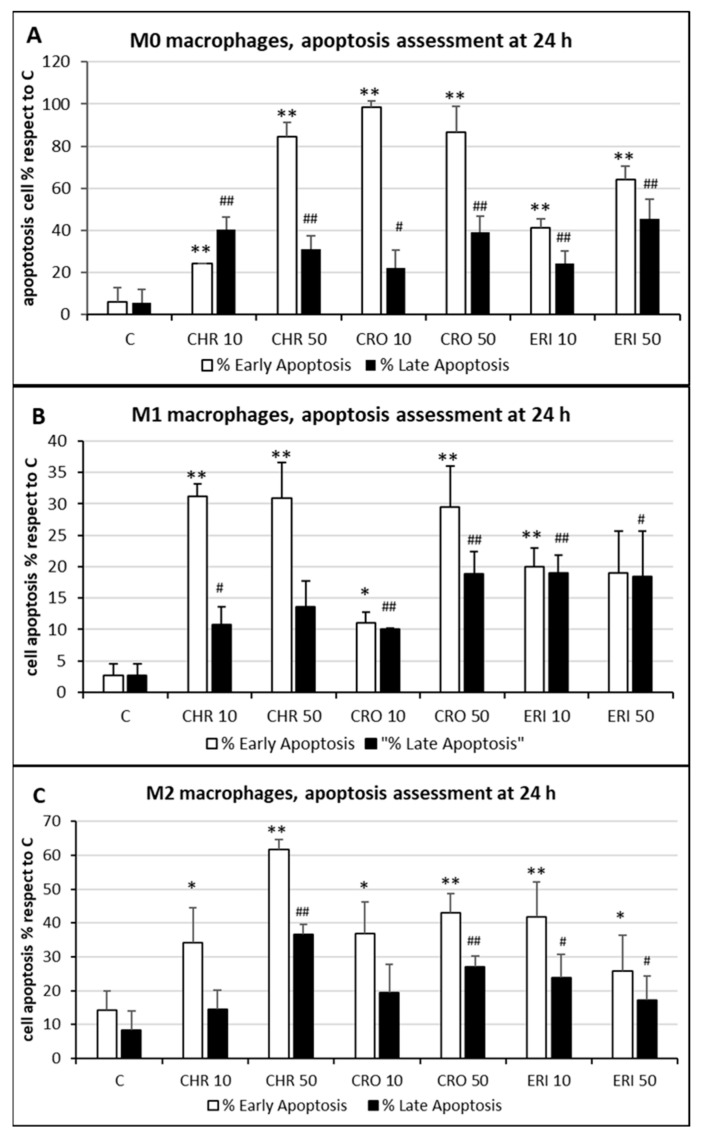
Cell apoptosis quantification. (**A**) The quantitative analysis of early (annexin-positive, white bars) and late (both annexin-positive and propidium iodide-positive, black bars) apoptotic M0 THP-1 cells treated with 50 and 10 μg/mL of CRO, CHR and ERI relative to the total number of cells observed by confocal microscopy after 24 h; results are the mean ± SD of counts from five microphotograph. Asterisks indicate significance in Tukey test (ANOVA for white bars *p* < 0.000001; Tukey vs. C, ** *p* < 0.005, respectively; ANOVA for black bars *p* < 0.0005, Tukey vs. C, ## *p* < 0.005, # *p* < 0.05, respectively). (**B**) The same analysis in pro-inflammatory M1 macrophages. ANOVA for white bars *p* < 0.00005; Tukey vs. C, ** *p* < 0.005, * *p* < 0.05, respectively; ANOVA for black bars *p* < 0.0001, Tukey vs. C, ## *p* < 0.005, # *p* < 0.05, respectively. (**C**) The same analysis in alternatively activated M2 macrophages. ANOVA for white bars *p* < 0.00001; Tukey vs. C, ** *p* < 0.005, * *p* < 0.05, respectively; ANOVA for black bars *p* < 0.00005, Tukey vs. C, ## *p* < 0.005, # *p* < 0.05, respectively.

In general, in all differentiated macrophages, exposure to CRO was not able to induce cell lysis, meaning that the high rates of cytotoxicity, measured by the alteration of energy metabolism through the MTT test at 24 h for the same fibre (Figure 3A–C), are the result of other death mechanisms. On the other hand, significant levels of cell lysis were observed after treatment with CHR or ERI. Indeed, in the case of ERI, our results confirm recent findings in the U937 monocyte cell line model, which also demonstrated the direct damage of cell membranes in contact with the fibres [39]. The level was higher for CHR compared to ERI, and this result may point towards multifactorial cytotoxic activity, with cell membrane alteration at least partially capable of inducing cell death. To test this hypothesis, cells were treated with the mineral fibres for 24 h and analysed by confocal microscopy using annexin/propidium iodide staining to both qualitatively (Figure 4) and quantitatively (Figure 5) assess their apoptotic state. Confocal microscopy analysis indicated that in all types of differentiated macrophages (Figure 4A, M0 macrophages; 4B, M1 macrophages; 4C, M2 macrophages), although at different rates, all fibres (Figure 4A–C: panels III-IV CRO, panels V-VI CHR and panels VII-VIII ERI, respectively) caused the significant induction of both early (only green positivity) and late apoptosis (concomitant green/red positivity) compared to control cells (Figure 4A–C: panels I-II in each figure). 

The quantitative assessment of the apoptotic state, obtained by counting and calculating the mean of only green and red–green cells in five different fields of confocal microscope images for each treatment, is shown in Figure 5. In M0 cells (panel A), CRO induced the highest levels of early signals of apoptosis at both concentrations tested (white bars, 98% and 86% for 10 and 50 μg/mL CRO, respectively, *p* < 0.005 vs. C, for both bars). Early apoptosis signals after exposure to CHR and ERI were nonetheless significant and dose-dependent, although lower than those after exposure to CRO (white bars, 24% and 84% for 10 and 50 μg/mL CHR, and 41% and 64% for 10 and 50 μg/mL ERI, respectively, *p* < 0.005 vs. C for all bars). In contrast, regardless of the concentration used, the number of late apoptotic cells after 24h of treatment was similar for all fibres (black bars, from ~20 to ~40%, *p* < 0.005 vs. C). These data, showing an increase in apoptotic levels for all fibres, are in agreement with the apoptosis assessment in THP-1 M0 cells in our previous work [9], which was performed with a shorter incubation time (8 h), indicating further progression of the apoptotic phenomenon in these cells over time. On the other hand, compared to M0 cells, M1 macrophages (panel B) showed slightly reduced levels of apoptosis. Indeed, in M1 cells, the highest rates of early apoptosis were 31% for both 10 and 50 μg/mL CHR (white bars, *p* < 0.005 vs. C for all), although all values of early apoptosis were found to be significant compared to control cells (~20% for 10 and 50 μg/mL ERI with *p* < 0.005 vs. C for both bars, 11% for 10 μg/mL CRO and 29% 50 μg/mL CRO with *p* < 0.05 and *p* < 0.005 vs. C, respectively). In addition, all mineral fibres induced late apoptosis to a certain degree (black bars), ranging from ~10% to ~19%, with the highest values measured for 10 and 50 μg/mL ERI and 50 μg/mL CRO (*p* < 0.05 vs. C for all bars). Finally, M2 macrophages (panel C) showed the highest rates of early apoptosis after CHR treatment (white bars, 37% and 62% for 10 and 50 μg/mL CHR, respectively, *p* < 0.005 vs. C for both) and a lower rate for CRO (37% and 43% for 10 and 50 μg/mL CRO, respectively, *p* < 0.005 vs. C for both) and ERI (41% and 26% for 10 and 50 μg/mL ERI, respectively, *p* < 0.05 vs. C). Regarding late apoptosis, in these cells, it was significantly increased only by the highest concentrations of CHR and CRO compared to control cells (black bars, 37% for 50 μg/mL CHR and 27% for 50 μg/mL CRO, respectively, *p* < 0.005 vs. C for both). Conversely, both concentrations of ERI appeared to induce significant levels of late apoptosis (24% and 17% for 10 and 50 μg/mL ERI, respectively, *p* < 0.05 vs. C for both).

Overall, our data indicate that the three types of fibres are able to induce significant damage, resulting in the alteration of energy metabolism, cell membrane lysis and/or apoptosis and likely macrophage activation through phagocytosis. The overall phenomena, together with the incapability of macrophages to completely digest engulfed fibres and the induction of apoptosis resistance in macrophages by the fibres through activation of mitochondrial NOX4 over time [15], lead to a state of prolonged local inflammation and the release of inflammatory cytokines prodromal to the onset of cancer [40,41]. Furthermore, macrophages internalising small fibres may survive in the short term and may act as carriers that transport particles towards the inner lung parenchyma and the mesothelium. The chronic inflammatory state caused by undigested fibres in activated macrophages at these new sites, as well as the direct interaction of the transported fibres with epithelial and mesothelial cells, will then set up a favourable microenvironment for cell transformation and carcinogenesis. 

### 2.3. ROS Production and DNA Damage

**Figure 6:**
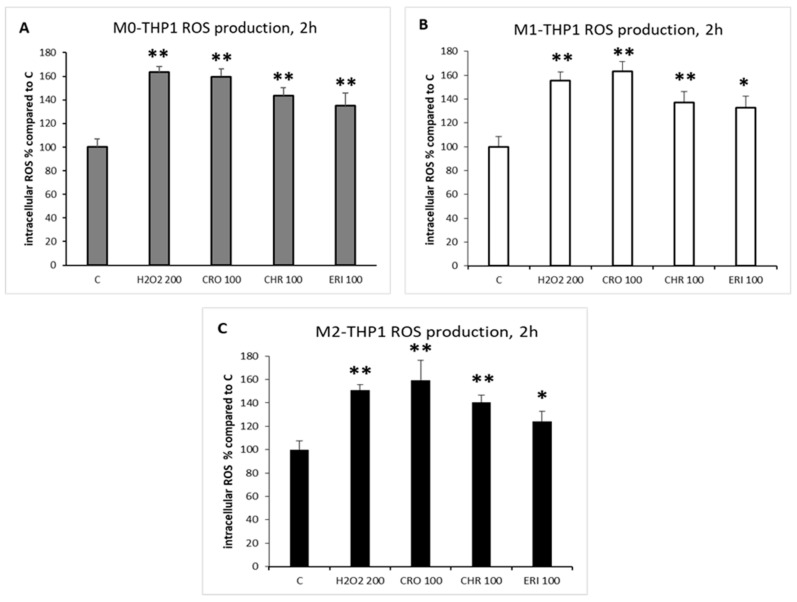
Intracellular ROS evaluation. (**A**) Intracellular ROS production measured by 2′,7′-dichlorodihydrofluorescein diacetate fluorometric analysis in M0 macrophages incubated for two hours in the presence or absence of 100 µg/mL of mineral fibres CRO, CHR and ERI. Cells stimulated with 200 µM H_2_O_2_ are the positive control. Results are expressed as percentages of ROS production compared to the untreated control (**C**) and are the mean ± SD of three experiments, in which each condition was tested eight times. Asterisks indicate significance in Tukey test (ANOVA *p* < 0.000001; Tukey vs. C, ** *p* < 0.0001). (**B**) Intracellular ROS production in pro-inflammatory M1 macrophages in the same conditions as (**A**). ANOVA *p* < 0.000001; Tukey vs. C, * *p* < 0.005, ** *p* < 0.0001, respectively. (**C**) Intracellular ROS production in alternatively activated M2 macrophages in the same conditions as (**A**). ANOVA *p* < 0.000001; Tukey vs. C, * *p* < 0.005, ** *p* < 0.0001, respectively.

In the lungs, lung-resident macrophages are key contributors to triggering and maintaining the inflammatory response to harmful stimuli [42]. Macrophage cells are known to undergo a respiratory burst following cellular activation and phagocytic events [43], thus modifying the oxidative state of the cells through the intracellular production of ROS. In addition to constituting a physiological response that is also very important for signal transduction [43], the ROS increase can be further exacerbated by the physical-chemical nature of the phagocytosed particles, which, in the case of the three mineral fibres, present several redox-active metals on their surfaces that are able to promote ROS generation (see results in Figure 1 and Table 1). Thus, significant oxidative stress is to be expected by their administration to living cells and organisms [44]. Specifically, to measure intracellular ROS production, the different types of macrophages, M0, M1 and M2 (Figure 6, panels A, B and C, respectively), were challenged with the mineral fibres for 2 h, and the ROS increase was compared to that in the untreated control cells (C) and to positive control cells stimulated by 200 μM H_2_O_2_. This time point was chosen because ROS production is a documented early signal of the inflammatory response [43] and because it was previously observed that further increases in ROS production in THP-1 cells after 2 h are not significant, remaining almost the same up to 4 h after the challenge with mineral fibres [9]. 

In all types of differentiated macrophages, the three mineral fibres were able to significantly increase intracellular ROS production after 2 h compared to the untreated control cells, with no significant differences among the three types of cells. The extent of this increase was comparable to that in the positive control (H_2_O_2_ stimulus, from 1.5-fold to 1.63-fold increase in all differentiated macrophages compared to C) for CRO and slightly lower but still significant if compared to untreated control cells for CHR and ERI. In particular, the exposure of M0, M1 and M2 macrophages to CRO, CHR and ERI fibres increased intracellular ROS production by ~1.6-fold, ~1.4-fold and 1.2–1.35-fold compared to untreated control cells (panels A–C), respectively. ERI showed a lower ability to increase intracellular ROS production in the three types of macrophages compared to the other two asbestos fibres. This lower cellular response is likely due to the different metal ion compositions of the fibres. In fact, CRO is able to release significant amounts of intracellular iron (see Figure 8B), and CHR releases several redox-active metals (i.e., Mg, Fe, Cr, Ni and Co, see Figure 8A–D). On the other hand, ERI hosts significantly lower amounts of redox-active metals. It was also found that ERI only releases Al from the surface of the fibres (dealumination) into the cytoplasm in quantifiable amounts (see Figure 8B) after 24 h. On the other hand, ERI is known for its cation-exchange capacity (CEC, [14]), which is able to affect intracellular Na^+^ and Ca^++^ concentrations in M0 macrophages [9]. Due to the CEC, Na^+^ is released from ERI fibres, and Ca^++^ is sequestered, resulting in a significant cytoplasmic rise in Na^+^ and a clearly measurable depletion of Ca^++^. It is well known that Ca^++^ is a fundamental stimulus for intracellular ROS production, both for cytoplasmic NADPH oxidase isoform activation and for mitochondria-mediated ROS production [45]. Thus, Ca^++^ depletion due to the CEC, together with the reduced release of redox-active metals from the surface of ERI fibres, explains the lower propensity of ERI to generate intracellular ROS compared to the other two asbestos fibres.

**Figure 7:**
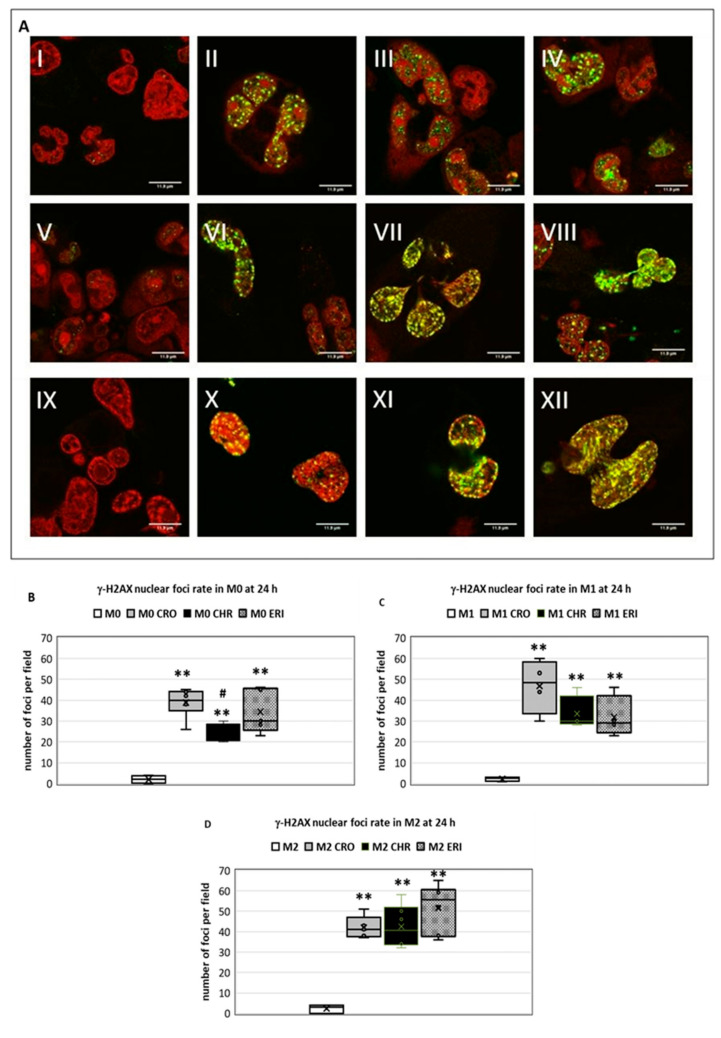
Quantification of genotoxic damage. (**A**) Visualisation by confocal microscopy analysis (4 × digital zoom) of double-strand breaks in DNA by γ-H2AX protein staining (green fluorescence), and DNA propidium iodide staining (red fluorescence) in differentiated macrophages after 24 h of incubation with 50 µg/mL fibres. Panels I–IV: M0-THP-1 macrophages; panels V–VIII: M1-THP-1 macrophages; panels IX-XII: M2 THP-1 macrophages. Panels I, V and IX: control; panels II, VI and X: CRO; panels III, VII and XI: CHR; panels IV, VIII and XII: ERI. The white bar represents 12 µm. (**B**) The quantitative analysis results of confocal microscopy acquisitions are expressed as the total number of double-strand breaks in the DNA of M0 THP-1 challenged with 50 µg/mL fibres and are the mean ± SD of five microphotographs. White bar: control; grey bar: CRO; black bar: CHR; dotted bar: ERI. Asterisks indicate significance in Tukey test (ANOVA *p* < 0.000005; Tukey vs. M0, ** *p* < 0.001, Tukey vs. M0 CRO # *p* < 0.001, respectively). (**C**) The same analysis as (**B**) in M1 THP-1 macrophages (ANOVA *p* < 0.0005, Tukey vs. M1, ** *p* < 0.001). (**D**) The same analysis as (**B**) in M2 THP-1 macrophages (ANOVA *p* < 0.00005, Tukey vs. M2, ** *p* < 0.001).

The increase in the oxidative state of the cells due to asbestos fibres contributes to the induction of various forms of DNA damage [46] with the subsequent onset of various malignancies, namely, diffuse malignant mesothelioma and lung cancer. In fact, exposure to asbestos fibres results in the appearance of DNA double-strand breaks (DSBs) in lung cells, particularly through the production of ROS (as quantified in Figure 6), which can reach the cell nucleus and attack the DNA backbone as well as the four DNA bases [47]. Thus, after 24 h of exposure to fibres, DNA DSBs (Figure 7) were qualitatively (panel A) and quantitatively (panel B) evaluated in M0, M1 and M2 cells by confocal microscopy through the staining of γ-H2AX foci. 

The ratio between the number of foci in treated cells compared to the number of foci in untreated cells (panel B) was then quantified. As expected, after exposure to mineral fibres, a significant rate of DSBs was found in the macrophages, regardless of the type of differentiation that they underwent, with no significant differences among the various fibre treatments. Specifically, the foci ratios measured in treated M0 cells compared to the untreated control (mean number of treated M0 foci/mean number control M0 foci) were ~17, 12 and 17.2 for CRO (black bars), CHR (grey bars) and ERI (white bars) (*p* < 0.001 vs. C for all bars, respectively), whereas in M1 macrophages, the ratio corresponded to 23.3, 21.6 and 20.5 for CRO, CHR and ERI, respectively (*p* < 0.001 vs. C for all bars). Finally, in M2 cells, the foci ratio amounted to 18, 18.2 and 22.1 for CRO, CHR and ERI, respectively, relative to untreated control cells (*p* < 0.0005 vs. C for all bars). 

The results clearly demonstrate that in the three types of macrophages, the mineral fibres showed different rates of toxicity and different proportions between plasma membrane lysis and apoptosis as causes of cell death (Figure 2, Figure 3 and Figure 4), with CRO favouring the induction of cell death by apoptosis and CHR and ERI inducing cell death by both cell lysis and apoptosis. When it comes to DNA damage, all three fibres showed very similar behaviours, with a significant number of DSB foci already formed at 24 h in all treatments. This confirms that the onset of cell genomic instability induced by the fibres takes place shortly after their inhalation and that there is no apparent difference between the genetic damage caused by the three classified carcinogens. 

### 2.4. Intracellular Dissolution of Fibres and Metal Release

The interaction of mineral fibres with lung cells involves a wide range of biological processes and cellular responses in which professional phagocytes (e.g., macrophages and neutrophils) play a leading role. In the first hours after contact, the mineral fibre is partially or completely engulfed by phagocytes and enclosed in intracellular phagosomes, which undergo fusion with lysosomal vesicles (acidic compartments filled with different types of hydrolases) in a process called ‘phagosome maturation’, leading to the degradation of the phagolysosome content. Evidence from in vivo and in vitro studies has shown that mineral fibres can undergo partial or total dissolution during phagocytosis [48,49]. This process is a function of the fibre biodurability (i.e., the resistance of the fibres to chemical/biochemical alteration). Indeed, recent in vitro acellular dissolution experiments showed that CRO, CHR and fibrous ERI display very different dissolution rates in the macrophage phagolysosome environment [48]. For a 0.25 μm thick fibre, the estimated time for complete dissolution of chrysotile from Balangero is 0.3 y, which is very short if compared to that of UICC standard crocidolite (66 y) and fibrous erionite from Jersey, USA (181 y). Because mineral fibres can host toxic metals in their structure, fast-dissolving fibres such as chrysotile can act as carriers that release their metal cargos into the extracellular and intracellular environments more quickly. Conversely, biodurable crocidolite releases its metals slowly. Fibrous erionite is a biodurable zeolite that has an ion-exchange capacity. It can therefore release extra-framework cations by ion exchange even if dissolution does not occur [44].

**Figure 8:**
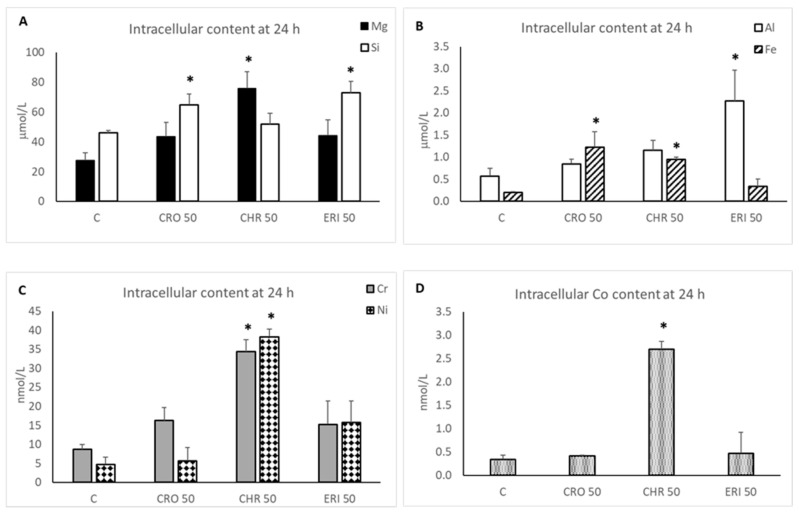
Intracellular silicic acid and toxic metal quantification. (**A**) Intracellular silicic acid content (white bars), measured by the Spectroquant*^®^* Silicate (Silicic Acid) Test, and Mg content (black bars), measured by inductively coupled plasma–mass spectrometry (ICP-MS), in M0 THP-1 macrophages incubated for 24 h with 50 µg/mL fibres. Results are the mean ± SD of two independent experiments performed in duplicate. Asterisks indicate significance in Tukey test (ANOVA for Si content *p* < 0.0005; Tukey vs. C, * *p* < 0.05, ANOVA for Mg content *p* < 0.05, Tukey vs. C * *p* < 0.05, respectively). (**B**) Intracellular Al content (white bars) and Fe content (striped bars) in M0 THP-1 cells in the same conditions as (**A**) measured by ICP-MS. ANOVA for Al *p* < 0.01; Tukey vs. C, * *p* < 0.05; ANOVA for Fe *p*< 0.001, Tukey vs. C * *p* < 0.05; respectively. (**C**) Intracellular Cr content (grey bars) and Ni content (dotted bars) in M0 THP-1 cells in the same conditions as (**A**) measured by ICP-MS. ANOVA for Cr *p* < 0.0005; Tukey vs. C, * *p* < 0.05; ANOVA for Ni *p* < 0.0001, Tukey vs. C, * *p* < 0.05. (**D**) Intracellular Co content (dotted bars) in M0 THP-1 cells in the same conditions as (**A**) measured by ICP-MS. ANOVA *p* < 0.00005; Tukey vs. C, * *p* < 0.0001.

To test this hypothesis and to measure the partial dissolution of the fibres inside macrophages at 24 h, polarised M0 THP-1 cells were treated with the three mineral fibres, and the intracellular content of silicic acid (as a measure of Si release in a soluble state) and the concentrations of selected trace elements (Mg, Al, Fe, Cr, Ni and Co) were detected (Figure 8). 

The basal intracellular soluble Si concentration in M0 THP-1 untreated macrophages was 46 μM (panel A, white bars), and only the CRO and ERI treatments induced significant increases of 1.41- and 1.59-fold compared to control cells, respectively (*p* < 0.001 vs. C for both). CHR did not show a measurable soluble Si increase after 24 h of treatment, and this could be due to the different dissolution mode of this fibre compared to CRO and ERI [48]. In fact, in the case of CHR, dissolution involves the brucite octahedral sheet [48,50], while the Si-centred tetrahedral sheet is biodurable [44]. These observations are confirmed by the fact that a significant increase in Mg intracellular content, originating from the dissolution of the brucite sheet of the mineral, was only measured in CHR-treated M0 THP1 macrophages (1.6-fold increase, *p* < 0.05) as compared to control cells (basal intracellular Mg concentration of 27.4 μM) and to CRO- or ERI-treated cells (panel A, black bars). The homeostasis of Mg, both intracellular and extracellular, is tightly regulated in the body due to the pleiotropic functions of this ion in hundreds of enzymatic activities, particularly those involved in the storage, transfer and utilisation of energy [51]. Thus, changes in the intracellular concentration of free Mg are linked to imbalances of energy metabolism, which were indeed measured in the MTT tests (see Figure 3), ion transport, signal transduction and, ultimately, cellular stress. These effects can contribute not only to the acute toxicity of CHR but also to the onset of chronic disorders since the alteration of these important cellular activities is also linked to epigenetic changes that affect the long-term physiology of cells [52]. Intracellular concentrations of Fe and Al (panel B) were also found in the micromolar range in untreated macrophages (0.2 and 0.5 μM, respectively). Furthermore, Fe was significantly increased in the cytoplasm of CRO- and CHR-treated cells (6.1- and 4.7-fold increases, respectively, white and striped bars, *p* < 0.05 for both), while Al was only significantly increased by ERI treatment (2.6-fold increase, *p* < 0.05) as compared to control cells. It is well known that both metals exert their toxic effects by creating an intracellular oxidative environment [53,54] that leads to cellular dysfunctions such as mitochondrial impairment, protein and lipid oxidation and DNA damage, which indeed were also observed in our mineral-fibre-treated cells (see Figure 3, Figure 6 and Figure 7). Thus, the significant release of these metal ions from the fibres helps to explain the increased ROS production and the onset of the oxidative environment that we observed in differentiated M0, M1 and M2 macrophages (Figure 6) in the presence of the three different mineral species. Finally, it is noteworthy that CHR was also able to release traces of other toxic elements, such as Cr, Ni (panel C, grey and diamond bars, respectively) and Co (panel D), significantly increasing the very low basal intracellular content of these elements, which were in the nanomolar concentration range in untreated M0 cells (8.6, 4.7 and 0.34 nM, respectively). The accumulation of these metals in the body, mainly in the lungs due to inhalation of polluted air, is related to the development of direct or indirect oxidative stress and cancer promotion, as reviewed in Valko et al. [55]. Furthermore, the toxicity of Ni and Cr has also been recently demonstrated in the induction of nuclear epigenetic changes such as an increase in DNA and histone methylation at specific chromatin regions with the consequent downregulation of tumour suppressors such as p16, a cell cycle regulator [56]. These findings explain how the release of these toxic metals promotes neoplastic cell transformation after inhalation. In CHR-treated macrophages, we measured a significant increase of 2.3-fold for intracellular Cr, 4.8-fold for Ni and 4.6-fold for Co as compared to control macrophages (*p* < 0.05 vs. C for all). These results agree with recent findings from Gualtieri et al. [44,48] in acellular in vitro dissolution tests. In fact, the dissolution of the chrysotile fibres involves the decomposition of the octahedral sheet with the leaching of Mg, Fe, Cr, Ni and Co. On the other hand, crocidolite is known to undergo slow dissolution with the formation of a nanometre-thick amorphous surface layer. Thus, the partial dissolution of crocidolite is responsible for the release of Fe in the CRO sample. The difference in the Fe content between Balangero chrysotile (1.4 wt% FeO and 0.2 wt% Fe_2_O_3_) and UICC crocidolite (17.4 wt% FeO and 17.9 wt% Fe_2_O_3_) accounts for the difference in the iron found in CHR-treated cells compared to CRO-treated cells. Once again, the results obtained for the Al content in ERI-treated macrophages confirm the previous acellular in vitro data.

Our results may help to explain why, despite the low biodurability and the supposed lower toxicity potential of chrysotile relative to the other two mineral fibres, this mineral is carcinogenic and represents a hazard, especially for subjects exposed in work environments [57,58]. Thus, in contrast to the other two mineral fibres, CRO and ERI, whose carcinogenicity can be ascribed to their persistence in the lungs for years, the adverse effects of CHR seem to also be due to the dissolution process itself, with the release of metal ions such as Mg, Fe, Cr, Ni and Co. Indeed, the sum of all of these metals will amount to a significant concentration of oxidative stress, promoting the presence of ions in the cytoplasm of the affected cells and causing the onset of cellular dysfunctions and possibly neoplastic transformation due to both genetic and epigenetic changes.

### 2.5. Inflammatory Response

**Figure 9:**
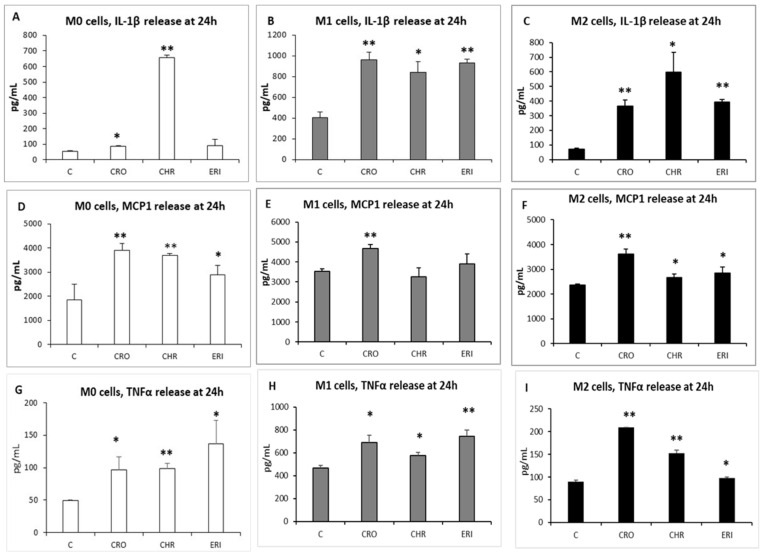
Extracellular release of inflammatory cytokines. (**A**) IL-1β release (in pg/mL) quantified by ELISA kit in M0 THP-1 macrophages incubated for 24 h with 50 µg/mL CRO, CHR and ERI. Results are the mean ± SD of two independent experiments performed in duplicate. Asterisks indicate significance in Tukey test (ANOVA *p* < 0.000001; Tukey vs. C, * *p* < 0.0005, ** *p* < 0.0001, respectively). (**B**) IL-1β release in M1 THP-1 macrophages in the same conditions as (**A**). ANOVA *p* < 0.000005; Tukey vs. C, * *p* < 0.001, ** *p* < 0.0001, respectively. (**C**) IL-1β release in M2 THP-1 macrophages in the same conditions as (**A**). ANOVA *p* < 0.00005; Tukey vs. C, * *p* < 0.001, ** *p* < 0.0001, respectively. (**D**) MCP-1 release (in ng/mL) quantified by ELISA kit in M0 THP-1 incubated as in (**A**). ANOVA *p* < 0.0001; Tukey vs. C, * *p* < 0.05, ** *p* < 0.005, respectively. (**E**) MCP-1 release in M1 THP-1 macrophages in the same conditions as (**A**). ANOVA *p* < 0.001; Tukey vs. C, ** *p* < 0.0005. (**F**) MCP-1 release in M2 THP-1 macrophages in the same conditions as (**A**). ANOVA *p* < 0.00005; Tukey vs. C, * *p* < 0.01, ** *p* < 0.0005, respectively. (**G**) TNF-α release (in pg/mL) quantified by ELISA kit in M0 THP-1 macrophages incubated in the same conditions as in (**A**). ANOVA *p* < 0.005; Tukey vs. C, * *p* < 0.05, ** *p* < 0.0005, respectively. (**H**) TNF-α release in M1 THP-1 macrophages in the same conditions as (**A**). ANOVA *p* < 0.00005; Tukey vs. C, * *p* < 0.005, ** *p* < 0.0005, respectively. (**I**) TNF-α release in M2 THP-1 macrophages in the same conditions as (**A**). ANOVA *p* < 0.00005; Tukey vs. C, * *p* < 0.001, ** *p* < 0.0001, respectively.

**Figure 10:**
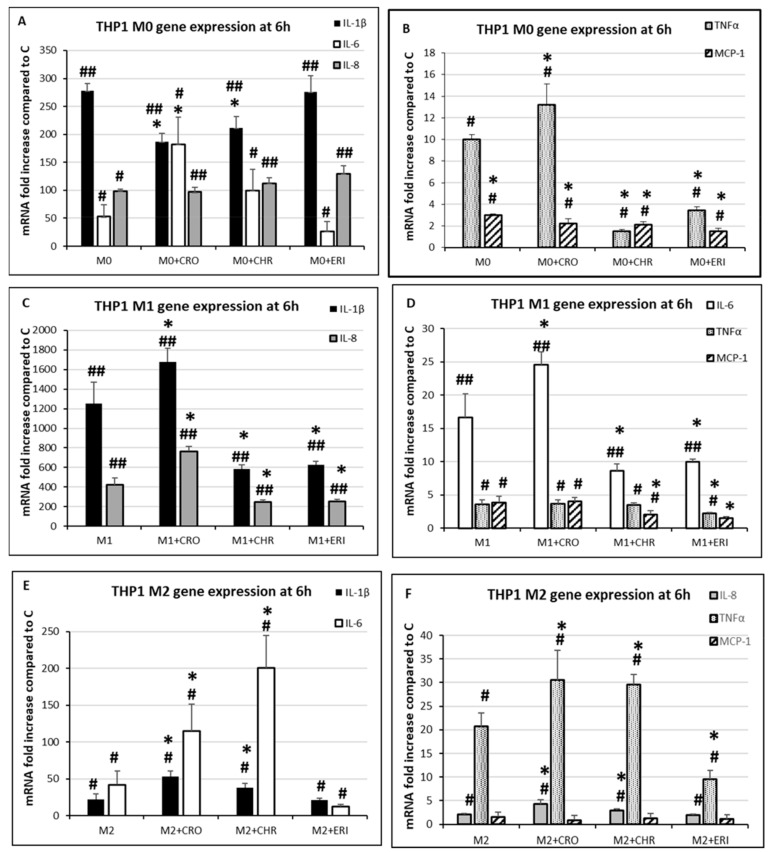
Gene expression of inflammatory mediators. (**A**) Gene expression of IL-1β, IL-6 and IL-8 measured by qPCR analysis after M0 THP-1 cell incubation for 6 h with 50 μg/mL mineral fibres (CRO, CHR and ERI). Data are normalised to the HPRT-1 housekeeping gene and expressed as mRNA fold increase compared to undifferentiated (control) THP-1 monocytes. Results are the mean ± SD of three experiments performed in triplicate. Asterisks indicate significance in Tukey test (IL-1β ANOVA *p* < 0.000001, IL-6 ANOVA *p* < 0.0005, IL-8 ANOVA *p* < 0.000001; Tukey vs. C # *p* < 0.05, ## *p* < 0.0001; Tukey vs. M0 * *p* < 0.05, respectively). (**B**) Gene expression of TNF-α and MCP-1 measured by qPCR analysis in M0 THP-1 cells in the same conditions as (**A**). TNF-α ANOVA *p* < 0.000001, MCP-1 ANOVA *p* < 0.00005, Tukey vs. C # *p* < 0.05, Tukey vs. M0 * *p* < 0.05, respectively. (**C**) Gene expression of IL-1β and IL-8 measured by qPCR analysis in M1 THP-1 cells in the same conditions as (**A**). IL-1β ANOVA *p* < 0.000001, IL-8 ANOVA *p* < 0.000001, Tukey vs. C ## *p* < 0.0001, Tukey vs. M1 * *p* < 0.05, respectively. (**D**) Gene expression of IL-6, TNF-α and MCP-1 measured by qPCR analysis in M1 THP-1 cells in the same conditions as (**A**). IL-6 ANOVA *p* < 0.000001 TNF-α ANOVA *p* < 0.0001, MCP-1 ANOVA *p* < 0.0005, Tukey vs. C # *p* < 0.05, C ## *p* < 0.0001, Tukey vs. M1 * *p* < 0.05, respectively. (**E**) Gene expression of IL-1β and IL-6 measured by qPCR analysis in M2 THP-1 cells in the same conditions as (**A**). IL-1β ANOVA *p* < 0.000005, IL-6 ANOVA *p* < 0.00005, Tukey vs. C # *p* < 0.05, Tukey vs. M2 * *p* < 0.05, respectively. (**F**) Gene expression of IL-8, TNF-α and MCP-1 measured by qPCR analysis in M2 THP-1 cells in the same conditions as (**A**). IL-8 ANOVA *p* < 0.0001 TNF-α ANOVA *p* < 0.000005, Tukey vs. C # *p* < 0.05, Tukey vs. M2 * *p* < 0.05, respectively.

Upon exposure to harmful stimuli, immune cells, especially macrophages, are responsible for both triggering and maintaining the inflammatory response [2]. For this reason, they have a fundamental role in the production of a number of cytokines that regulate the immune response in both acute and chronic conditions. The production of inflammatory cytokines is stimulated by the activation of the scavenger receptor A family upon the engagement and phagocytosis of foreign particles [59] and is also sustained by the respiratory burst, mainly through the generation of H_2_O_2_. The latter is known to activate NF-κB and MAPK signalling pathways for the upregulation of gene transcription [43], as well as to activate the NLRP3 inflammasome for the cleavage and maturation of cytoplasmically stored cytokines prior to their release in the extracellular milieu [16]. Thus, the result of this activation leads to the production of major inflammatory mediators [60]. According to the well-known fact that asbestos leads to chronic inflammation in the lung tissue, and since in our experimental conditions, we observed phagocytic events (Figure 1 and Figure 2) and ROS production (Figure 6) in all three types of differentiated macrophages after only 24 h, the extracellular release of cytokines and the gene expression profile were evaluated by ELISA (Figure 9) and qPCR (Figure 10) tests, respectively, in M0, M1 and M2 cells in contact with the fibres. The reason to investigate both parameters is that while the rapid release of cytokines in the extracellular milieu indicates an acute response to the inflammatory stimulus, in the case of the mineral fibres, conversely, changes in the same cytokines at the transcriptional level may point to an exacerbated inflammatory response that persists over time and contributes to the onset of a chronic state of inflammation.

The pro-inflammatory protein IL-1β is particularly well known for its involvement in several cellular activities, such as cell proliferation, differentiation and apoptosis [61]. In M0 macrophages (Figure 9A), basal IL-1β release amounted to 54.95 ± 4.77 pg/mL, which was increased by 1.6-fold by both CRO and ERI treatments and by 11.9-fold by CHR (*p* < 0.0005 and *p* < 0.0001 vs. C for CRO and CHR, respectively). Due to their pro-inflammatory role, M1 cells (Figure 9B) showed an overall higher basal production of this cytokine, which amounted to 406.5 ± 55 pg/mL of IL-1β in the untreated cells compared to M0 macrophages (7.4-fold increase compared to control M0 cells). Its release was further significantly elevated by all mineral fibres, exhibiting 2.4-fold and 2.3-fold increases in CRO- and ERI-treated cells (*p* < 0.0001 vs. C for both bars), respectively, and a 2.1-fold increase in CHR-treated cells (*p* < 0.001 vs. C). Regarding M2 macrophages (Figure 9C), the IL-1β quantified in the control cells was more similar to the release observed in M0 cells, and it amounted to 71.56 ± 6.45 pg/mL in the cellular medium (1.3-fold increase compared to control M0 cells). Again, its release was notably increased in all M2 cells in contact with the fibres, showing 5.1- and 5.5-fold increases for CRO and ERI, respectively (*p* < 0.0001 vs. C for both bars) and an 8.4-fold increase for CHR treatment (*p* < 0.001 vs. C). These data show that the three fibres induce very similar amounts of the pro-inflammatory cytokine IL-1β in M1 polarised macrophages. In M0 and M2 cells, CHR actually showed a much more relevant increase compared to the other two fibres, indicating that this fibre species can trigger a significantly stronger acute pro-inflammatory response after inhalation compared to CRO and ERI. Indeed, a significant IL-1β increase after CHR treatment has also been reported in vivo in a mouse model of CHR inhalation, where this cytokine was one of the highly expressed markers in the lungs of treated animals [62], corroborating the hypothesis that CHR also exerts a strong acute inflammatory effect through the significant intracellular release of toxic metals, as we observed after only 24 h (Figure 8). IL-1β overproduction by inflammatory cells is a signal that promotes both carcinogenesis and tumour growth during chronic inflammation thanks to the stimulation of cell proliferation and anti-apoptotic pathways, and it also acts as an angiogenic factor [63]. Thus, since CRO, CHR and ERI are able to stimulate the release of this cytokine not only in M0 and M1 macrophages but also, surprisingly, in the M2 phenotype, and knowing that these fibres persist for a long time in the lungs of exposed subjects (several months for CHR and years for CRO and ERI), it is reasonable to infer that chronically fibre-stimulated macrophages play a fundamental role in the carcinogenicity of asbestos and erionite fibres through the production of inflammatory cytokines that promote and maintain neoplastic cell transformation.

Another important cytokine involved in the inflammatory response is monocyte chemoattractant protein-1 (MCP-1), one of the key chemokines responsible for the regulation of migration and infiltration of monocytes/macrophages during the immune response [64]. For this reason, we analysed MCP-1 production in all three types of polarised macrophages in the presence or absence of the investigated fibres. MCP-1 production was already very high in untreated M0, M1 and M2 macrophages. Nevertheless, it significantly increased with the fibre treatment in all differentiated macrophages. Indeed, in M0 cells (Figure 9, panel D), the levels of MCP-1 (1862.7 ± 631 pg/mL) significantly increased by 2.1-fold and 1.99-fold with CRO and CHR, respectively (*p* < 0.005 vs. C for both bars) and 1.55-fold with ERI (*p* < 0.05 vs. C). In M1 macrophages (Figure 9, panel E), the basal levels of MCP-1 released in the medium were higher than those of M0 control cells (1.9-fold increase), and they were significantly increased after exposure to CRO (1.33-fold, *p* < 0.0005 vs. C). In contrast, in M2 macrophages (Figure 9, panel F), basal MCP-1 release showed only a slight increase (1.3-fold) compared to M0 control cells. Furthermore, in M2 cells exposed to CRO and ERI fibres, the release of this chemokine was further significantly increased by 1.5- and 1.2-fold (*p* < 0.0005 and *p* < 0.01 vs. C, respectively). In general, it is possible to conclude that CRO is invariably the most effective in stimulating the release of this chemotactic protein in the three types of differentiated macrophages compared to the other two fibres. For MCP-1, as in the case of IL1β, the production of this chemokine has important consequences in the onset and progression of cancers other than those due to the development of the inflammatory process [65]. In fact, MCP-1 production in the tumour microenvironment has been linked to a change in tumour-associated macrophages (TAMs) to an immunosuppressive phenotype, contributing to cancer cell proliferation and immunotherapy resistance [66]. As a matter of fact, this chemokine production also has an important role in mesothelioma development [67]. With this premise, our data demonstrate that the significant production of this chemokine by all three types of fibre-stimulated macrophages may contribute to the onset of conditions that are favourable to cancer progression in the lung because the release of this chemoattractant factor is able to suppress the immune system’s ability to fight tumour cell proliferation in the long term.

TNF-α is also a well-recognised cytokine involved in the progression of inflammation and fibrosis in lung respiratory diseases [68]. Indeed, the severity of the disease often correlates with TNF-α overexpression and release. Thus, this cytokine was also quantified in the cellular media of the three types of polarised macrophages incubated with the three fibres for 24 h.

The results (Figure 9G–I) show that all of the fibres were able to significantly increase TNF-α production after only 24 h of incubation in the three types of macrophages. In particular, in unstimulated M0 macrophages (panel G), the basal cytokine production amounted to 49.7 ± 0.3 pg/mL, and this further increased by 1.9-fold in the presence of both CRO and CHR (*p* < 0.05 and *p* < 0.0005 vs. C, respectively) and by 2.7-fold by ERI (*p* < 0.05 vs. C). In M1 cells (panel H), the basal release of the cytokine in unstimulated cells was significantly higher than in M0 control cells (9.4-fold). The fibre treatment further increased this production by 1.5-fold and 1.24-fold for CRO and CHR, respectively (*p* < 0.005 vs. C for both), and by 1.6-fold for ERI (*p* < 0.0005). Furthermore, in M2 cells, an increase in basal TNF-α production in unstimulated cells, as compared to M0 control cells, was also observed (1.8-fold). This production further increased by 2.3-, 1.7- and 1.1-fold in the presence of CRO, CHR and ERI, respectively (*p* < 0.0001 vs. C for the first two and *p* < 0.001 for the third). Once again, this cytokine, acting as a paracrine and autocrine growth factor, was demonstrated to have a role in tumour initiation, promotion, invasion and angiogenesis [69]. Thus, the significant TNF-α production measured in all three types of differentiated macrophages stimulated by the fibres once again shows the pro-tumour activity of these cells when recruited during the inflammatory response.

Our data confirm that all of the fibres cause significant production of well-known inflammatory and chemotactic cytokines in all macrophage phenotypes, with some differences among the various fibres. These results also indicate that after immunosuppressive M2 macrophages are recruited to the site of inflammation in response to mineral inhalation, the inflammatory response cannot be quenched since these cell types will also contribute to the production of pro-inflammatory factors, leading to the chronicisation of the phenomenon. Since the production of these cytokines is also clearly involved in cancer progression [41], these results confirm the hazard of all three mineral fibres investigated in this work.

Finally, we also analysed the gene expression profiles of interleukin-6 (IL-6) and interleukin-8 (IL-8), two other genes that are usually overexpressed in macrophages challenged with harmful stimuli and that are actively involved in the inflammatory response [70,71], together with the same inflammatory factors that were previously quantified by the ELISA tests in the different types of macrophages, displayed in Figure 9 (namely, IL-1β, MCP-1 and TNF-α). Both IL-6 and IL-8 were investigated because they are linked to detrimental effects of chronic inflammation. IL-6 sustains continuous monocyte recruitment to the site of inflammation, collagen synthesis and the anti-apoptotic effects of immune cells [70], while IL-8 displays strong chemoattractant activity, mitogenic and angiogenic effects, collagen synthesis and tumour growth promotion [71]. On the other hand, the reason to investigate the cytokine release of IL-1β, MCP-1 and TNF-α (Figure 9) together with the relative mRNA production (Figure 10) is due to the fact that the synthesis and storage of these pro-inflammatory factors are already upregulated during M0 polarisation as compared to untreated THP-1 monocytes. Thus, the cellular response to asbestos treatment could be due to either (i) the neo-synthesis of cytokines from stabilised cytoplasmic mRNAs and their release in the extracellular milieu, (ii) an increase in mRNA transcription by the control of gene expression at the DNA level or (iii) a combination of the two processes [72]. Thus, to investigate the regulation of IL-1β, MCP-1 and TNF-α release stimulated by the fibres in differentiated THP-1 macrophages, their gene expression was investigated as well (Figure 10) at the 6 h time point. This time point was chosen because cells in contact with the mineral fibres are still alive but have perceived the inflammatory stimulus by phagocytosis and/or plasma membrane contact and have already shown a respiratory burst that produces enough ROS to activate the necessary signal transduction pathways (see Figure 2, Figure 6 and Figure S1).

In M0 THP-1 macrophages, the mRNAs of all investigated cytokines (Figure 10A: IL-1β, IL-6 and IL-8; Figure 10B: MCP-1 and TNFα) were significantly increased as compared to the undifferentiated control THP-1 monocytes in the absence of the fibres (IL-1β, 278-fold increase; IL-6, 53-fold increase; IL-8, 99-fold increase; MCP-1, 3-fold increase; and TNF-α, 10-fold increase). This means that, in M0 macrophages, the mRNA transcripts of the major inflammatory cytokines are already available for immediate translation in the cytoplasm upon receiving an inflammatory stimulus. Conversely, regarding the further increase in mRNA expression upon fibre treatment of M0 cells compared to untreated M0 cells, we observed different behaviours depending on the measured cytokine. In the case of IL-1β, significant inhibition of mRNA synthesis was observed for CRO and CHR treatment (panel A, black bars, 37% and 24% decrease, *p* < 0.05 vs. M0) but not for ERI. On the contrary, IL-6 mRNA (panel A, white bars) showed a significant increase only in the presence of CRO (3.4-fold, *p* < 0.05 vs. M0) compared to untreated M0 cells, while IL-8 expression was always similar to untreated control M0 cells in all asbestos fibre-treated samples (panel A, grey bars). TNF-α mRNA was also found to be increased only in CRO-treated M0 cells (panel B, dotted bars, 1.3-fold, *p* < 0.05 vs. M0), while MCP-1 mRNA was not affected by treatment with any of the fibres compared to M0 cells (panel B, striped bars). These results indicate that in M0 cells, the significant cytokine production and release upon the addition of mineral fibres (Figure 9) are mainly due to protein synthesis from pre-existing cytoplasmic mRNA transcripts rather than the upregulation of transcription at the gene level, except for CRO, for which increases in IL-6 and TNF-α transcripts were also observed. Even in M1 THP-1 macrophages, the mRNAs of all of the investigated cytokines were significantly increased as compared to undifferentiated THP-1 monocytes and thus immediately available for translation if needed (Figure 10 C: IL-1β, 1250-fold increase; IL-8, 421-fold increase; Figure 10 D: IL-6, 17-fold increase; MCP-1, 3.9-fold increase; and TNFα, 3.6-fold increase). Moreover, a further increase in the expression of IL-1β, IL-6 and IL-8 cytokines in the M1 phenotype cells was observed upon CRO treatment. In particular, in the case of IL-1β mRNA (black bars), CRO treatment increased the synthesis by 1.3-fold (*p* < 0.05 vs. M1), while CHR and ERI inhibited the mRNA synthesis of this cytokine by 54% and 50%, respectively (*p* < 0.05 vs. M1 for both), although this reduction at the mRNA level did not affect the IL-1β cytokine release measured at 24 h, which indeed was relevant for all three types of fibres (Figure 9B). Regarding IL-8 mRNA expression (grey bars), CRO increased its synthesis by 1.8-fold (*p* < 0.05 vs. M1), while CHR and ERI diminished it by 50% in both cases (*p* < 0.05 vs. M1). In the case of IL-6 (white bars), again, its mRNA was increased by 1.5-fold by CRO treatment (*p* < 0.05 vs. M1), while CHR and ERI inhibited its synthesis by 40% in both cases (*p* < 0.05 vs. M1). Furthermore, in M1 cells, none of the fibre treatments was able to further increase the mRNA synthesis of TNF-α (dotted bars) or MCP-1 (striped bars) as compared to the untreated control M1 macrophages. Finally, mRNA expression analysis in differentiated M2 THP-1 macrophages (Figure 10E,F) once again revealed that the expression levels of IL-1β, IL-6, IL-8 and TNF-α were significantly increased compared to undifferentiated THP-1 monocytes (22-fold, 42-fold, 2-fold and 21-fold, respectively). Moreover, this mRNA production was further increased by both CRO and CHR treatment but not by ERI. Specifically, CRO and CHR increased IL-1β mRNA (panel E, black bars) by 2.4-fold and 1.7-fold (*p* < 0.05 vs. M2), IL-6 (panel E, white bars) by 2.7-fold and 4.8-fold (*p* < 0.05 vs. M2), IL-8 (panel F, grey bars) by 2.1-fold and 1.5-fold (*p* < 0.05 vs. M2), and TNF-α (panel F, dotted bars) by 1.5-fold and 1.4-fold, respectively (*p* < 0.05 vs. M2). Finally, in M2 cells, MCP-1 mRNA expression (panel F, striped bars) did not significantly differ from that of undifferentiated THP-1 monocytes in either the presence or absence of fibres. Noteworthy is the increase in the expression of pro-inflammatory cytokines, especially IL-6 and IL-8, in these cell types. Indeed, since this macrophage phenotype should appear at the site of inflammation at a later stage to help resolve the inflammatory state, the fact that M2 cells instead produce IL-6 and IL-8 in the presence of CRO and CHR seems to demonstrate the opposite, as these cytokines stimulate the chronicisation of inflammation, eventually promoting fibrosis and cancer cell growth.

These intriguing results (Figure 9 and Figure 10) suggest that the three macrophage phenotypes are able to respond to the detrimental stimuli of mineral fibres through the release of inflammatory mediators such as IL-1β, MCP-1 and TNF-α, mainly synthesised from pre-existing mRNA transcripts in the cytoplasm of the differentiated macrophages, with a minor contribution from upregulated transcription at the gene level. Notably, CRO seems to be able to also induce the upregulation of gene transcription of TNF-α in M0, M1 and M2 macrophages and of IL-1β, IL-6 and IL-8 in M1 and M2 fibre-stimulated macrophages. CHR is also able to partially induce this response at the transcriptional level by increasing IL-1β, IL-6, TNF-α and IL-8 only in M2 macrophages, while ERI never showed an upregulating effect at the transcriptional level of the investigated cytokines. As mentioned previously, there is a measurable decrease in Ca^2+^, which acts as an intracellular second messenger, in ERI-stimulated macrophages in the hours immediately after the addition of the fibres [9], and this could contribute to the lack of sufficient transduction signals able to induce an effect at the regulatory regions of cytokine genes in the nucleus in the first hours of stimulation. Thus, ERI inhalation could create a milder chronic inflammatory state in the lungs relative to that induced by CRO and CHR, and its tumorigenic effect may be more associated with the DNA-damaging properties of the fibres (see Figure 6) and their high biodurability in organic fluids [48]. Quite surprisingly, M2 macrophages, which are usually associated with a more anti-inflammatory phenotype, instead showed significant production of pro-inflammatory cytokines in the presence of the three types of fibres, probably contributing to the enhancement rather than the mitigation of the inflammatory state of the tissue. This may reasonably occur in the lungs of mineral-exposed subjects due to the persistence and biodurability of the mineral fibres, which undergo cycles of in situ ingestion and re-ingestion by phagocytic cells for months, as in the case of chrysotile, or for years, as in the case of crocidolite and erionite. Furthermore, the observed behaviour in the presence of the mineral fibres confirms that the distinction between the M1 and M2 phenotypes of macrophages is not so clear-cut and that these cells may show more fluid behaviour depending on the stimuli encountered and the extracellular milieu of the tissues to which they are recruited, with certain phenotypes exacerbating the inflammatory processes despite being initially recruited to obtain the opposite effect.

## 3. Materials and Methods

### 3.1. Chemicals

All reagents were acquired from SIGMA-ALDRICH (Milan, Italy), unless otherwise stated.

### 3.2. Mineral Fibres

The following mineral fibres were selected for the study: (i) UICC standard crocidolite (South Africa, NB #4173-111-3), (ii) chrysotile from Balangero (Turin, Italy), (iii) fibrous erionite-Na from Jersey (Nevada, USA). The morphological and morphometric characteristics of the three mineral fibres used in this study were described in detail in a previous work [9]. Additional information on the three mineral fibres can be found in the Supplementary Materials [73,74,75].

### 3.3. Cell Culture

The human monocytic cell line THP-1 used in this study was obtained from the American Type Culture Collection (LGC Standards srl, Milan, Italy). Cells were cultured at 37 °C in a humidified 5% CO2 atmosphere in RPMI-1640 with 2 mM L-glutamine (Euroclone, Milan, Italy) supplemented with 10% FBS (Euroclone), 50 μM β-mercaptoethanol and penicillin/streptomycin as antibiotics (Corning Inc, Corning, NY, USA). To evaluate the biological responses of differentiated macrophages in contact with mineral fibres, THP-1 monocytes were induced to polarise into non-activated M0 macrophages by adding 20 ng/mL phorbol-12-myristate 13-acetate (PMA, PeproTech EC, London, UK) to the culture medium. After 48 h, non-adherent cells were removed, and M0 macrophages were either employed for experiments or further polarised to pro-inflammatory M1 macrophages by treatment with 20 ng/mL interferon-gamma (IFNγ) and 200 ng/mL lipopolysaccharide (LPS) for 24 h or to alternatively activated M2 macrophages by using 20 ng/mL interleukin 4 (IL-4, PeproTech EC, London, UK) for 24 h [76,77].

### 3.4. Cell–Fibre Interaction Imaging and Fibre Surface Characterisation 

Micro-Raman spectroscopy was used for the detection and identification of the three different types of mineral fibres in differentiated M0, M1 and M2 macrophages. THP-1 monocytes were seeded at 300,000 cells/well in 6-well plates containing a 24 × 24 mm glass coverslip. Briefly, a 200 μL drop containing 300,000 cells was seeded on each coverslip, and after 2 h at 37 °C, the rest of the medium was added to the wells of the 6-well plate before the macrophages were differentiated as described above. Following their incubation with 50 µg/mL of fibres for 24 h, the cells were washed twice with PBS and then fixed with 4% paraformaldehyde in PBS at 4 °C. After 2 h, the glass coverslips were washed three times with sterile deionised water, and once the paraformaldehyde was completely removed, the coverslips were dried under a laminar flow hood. Micro-Raman measurements were performed with a HORIBA Jobin Yvon LabRam (HORIBA Scientific, Kyoto, Japan) confocal micro-spectrometer (300 mm focal length) with an integrated Olympus BX40 microscope with 4×, 10×, 50× ULWD and 100× objectives, 1800 grooves/mm grating, an XY motorised stage and a Peltier cooled silicon CCD. The Raman signals of the fibres were acquired in two spectral ranges: the lattice and internal vibrational modes were collected in the low-wavenumber spectral range (120–1200 cm**^−^**^1^) by using a He-Ne 632.8 nm laser line as the excitation source; the OH stretching signals were detected in the high-wavenumber spectral range (2700–4000 cm**^−^**^1^) by using a frequency-doubled Nd:YAG 473.1 nm laser line to distinguish the mineral phase among varieties whose spectra are similar in the low range. The system was calibrated with the 520.6 cm**^−^**^1^ Raman peak of silicon in the low-wavenumber range and with the emission lines of a gas lamp in the high-wavenumber range. The spectral resolution was ~2 cm**^−^**^1^ with the 632.8 nm line and ~4 cm**^−^**^1^ with the 473.1 nm line. The minimum spatial resolution was ~1 μm using the 100× objective. To avoid heating effects, density filters were used to reduce the laser power. The typical accumulation time was 60 s repeated 10 times. Data analysis was performed with the LabSpec 5 software. The fluorescence background was removed by subtracting a polynomial curve as the baseline. The Raman signals were deconvoluted with Gaussian–Lorentzian curves to determine the peak parameters and identify the mineralogical phases.

### 3.5. Cell Viability with Calcein Green-AM

In order to measure the short-term cytotoxicity of mineral fibres, THP-1 monocytes were seeded in eight-well Lab-Teck chambered slides (Nalge Nunc Int., Naperville, IL, USA) at 75,000 cells/well and differentiated into the three types of macrophages. After incubation with 50 µg/mL of fibres for 4 h, the macrophages were stained with 2 µM calcein green-AM (Life Technologies, Milan, Italy) following the manufacturer’s instructions. To assess cell viability, confocal microscopy images were obtained using a Leica TCS SL confocal microscope equipped with an HCX PL APO CS 20.0× objective (Leica Microsystems, Wetzlar, Germany). Finally, images of living cells (2.5 × digital zoom) were acquired in single stacks, both in phase-contrast mode and in fluorescence mode, acquiring the green fluorescence of calcein green-positive cells in the emission range of 500–550 nm.

### 3.6. Cytotoxicity Assessment by MTT and LDH

To assess the cytotoxic effect of the exposure to mineral fibres on M0, M1 and M2 macrophages, THP-1 monocytes were seeded in quadruplicate at 50,000 cells/well in 96-well plates and induced to differentiate according to the aforementioned treatments. Subsequently, the three different types of mineral fibres (10 µg/mL, 50 µg/mL and 100 µg/mL final concentrations) were added to each well, and the plates were incubated for 24 h at 37 °C. At the end of the experiments, cell viability was evaluated by the MTT assay (0.5 mg/mL final concentration) as previously reported in Pozzolini et al. [78]. Plasma membrane perturbation, which can lead to cell lysis, was quantified by measuring LDH release in the cell media through the quantification of enzymatic activity. Briefly, THP-1 cells were seeded in quadruplicate on 96-well plates, and the experiment was performed as described for the MTT assay. After 24 h of incubation with or without the different fibres, the LDH release in the cell media was measured by transferring 100 μL of cell medium from each well into wells of a new 96-well plate and adding 100 μL of assay buffer (686 μM iodonitrotetrazolium chloride, 291 μM 1-methoxyphenazine methosulphate, 1.35 mM μ-NAD and 55.5 mM lithium L-lactate in 200 mM TRIS, pH 8.2). The plate was then incubated at room temperature for 30 min, and then the reaction was blocked by adding 50 μL of 1 M acetic acid. The absorbance was read at 490 nm in a plate reader (BMG Labtech, Ortenberg, Germany). Data are the means ± SD of three independent experiments performed in quadruplicate. 

### 3.7. Apoptosis Detection by Confocal Microscopy

The level of cell apoptosis was assessed as previously described in Scarfì et al. [79] by employing a Leica TCS SL confocal microscope with argon/He-Ne laser sources and an HCX PL APO CS 20.0 × objective. For confocal microscopy imaging, THP-1 monocytes were plated in eight-well Lab-Teck chambered slides at a density of 75,000 cells/well, and after their polarisation, M0, M1 or M2 macrophages were incubated with the mineral fibres (10 µg/mL or 50 µg/mL) for 24 h at 37 °C. Subsequently, cells were stained using the Annexin V, FITC Apoptosis Detection Kit (Dojindo EU, Munich, GmbH) according to the manufacturer’s instructions. The acquired images (2.5 × digital zoom) were then obtained in phase-contrast mode and in fluorescence mode, acquiring the green fluorescence of annexin-positive cells and the red fluorescence of propidium iodide–positive cells (emission range of 500–550 nm and 600–670 nm, respectively) in single stacks.

### 3.8. ROS Intracellular Detection

After THP-1 monocytes were seeded in 96-well plates at a density of 50,000 cells/well and induced to polarise into the different types of macrophages, the assay was performed as described in De La Fuente et al. [80]. Briefly, after being washed once with HBSS, cells were incubated for 45 min at 37 °C with 10 µM 2**′**,7**′**-dichlorodihydrofluorescein diacetate dye in HBSS (Life Technologies) and then washed with HBSS to remove excess dye. ROS production in extracellular HBSS was evaluated after 2 h of exposure to 50 µg/mL or 100 µg/mL of mineral fibres at 37 °C, while the positive control was obtained with 200 µM H_2_O_2_. Then, the plates were read on a FLUOstar**^®^** Omega multi-mode microplate reader (BMG Labtech) with 485/520 excitation/emission wavelengths. Data are the means ± SD of two independent experiments, in which each condition was tested eight times.

### 3.9. Evaluation of DNA Damage

Since mineral fibres are known to induce genotoxic damage, the double-strand breaks in DNA were analysed by evaluating nuclear γ-H2AX histone foci. THP-1 cells were seeded in eight-well Lab-Teck chambered slides at 75,000 cells/well, and the differentiated macrophages were incubated with 50 µg/mL of fibres. After 24 h, cells were stained with anti-gamma H2AX antibody (Abcam, Cambridge, UK), while the nucleus was coloured with 2 µg/mL propidium iodide. Images were acquired with a Leica TCS SL confocal microscope with an HCX PL APO CS 63.0 × 1.40 oil objective. Nuclei images (4.0 × digital zoom) were acquired, in which the fluorescent foci of double-strand breaks in DNA are green and propidium iodide-positive nuclei are red (excitation at 488 nm for both and emission range of 500–550 nm and 600–670 nm, respectively).

### 3.10. Intracellular Silicate Quantification

Asbestos is a naturally occurring silicate mineral. Thus, the intracellular levels of silicic acid released by the fibres during phagocytosis were evaluated by seeding THP-1 cells in 12-well plates at 200,000 cells/well and subsequently differentiated into M0 macrophages as previously described. M0 cells were then treated with 50 µg/mL of mineral fibres for 24 h. Afterwards, the intracellular soluble silicates were quantified as described in Scarfì et al. [81]. Briefly, cells were washed twice with 0.9% NaCl, and their content was extracted with 1 mM perchloric acid. The extract was then centrifuged at 12000× *g* in a microfuge, and silicic acid in the supernatant was quantified using the Spectroquant**^®^** Silicate (Silicic Acid) Test (Merck KGaA, Darmstadt, Germany) according to the manufacturer’s instructions. Absorbance was measured at 650 nm using a Beckman spectrophotometer (DU 640), and the silicic acid concentration was obtained by comparison with a calibration curve based on different concentrations of sodium metasilicate (5–0.1 µg/mL). Data are the means ± SD of three independent experiments performed in duplicate.

### 3.11. Intracellular Trace Metal Quantification

The concentration of metals was determined in the cell lysate of M0 THP-1 cells treated with mineral fibres. Specifically, THP-1 cells were seeded in 10 cm plates at 3 × 10^6^ cells/plate, and then, following their polarisation to M0 cells as previously described, they were incubated with CRO, CHR and ERI at a concentration of 50 µg/mL for 24 h. After 24 h of exposure to the fibres, 3 mL of 1 mM perchloric acid (PCA) was added to the M0 THP-1 cells to induce lysis. The cell lysate was collected and centrifuged at maximum speed for 10 min to remove debris and fibres. Concentrations of Al, Fe, Mg, Ni, Co and Cr in cell lysate were determined using a Thermo Scientific iCAP™ TQ Inductively Coupled Plasma–Mass Spectrometer (ICP-MS). The iCAP TQ ICP-MS was configured to ensure the detection of analytes even at low concentrations and with small amounts of sample. Prior to measurements, plasma and interface settings were automatically tuned using dedicated Thermo Scientific software. The analyses were conducted with the application of TQ-O_2_ and SQ-KED measurement modes. TQ-O_2_ is triple quadrupole mode with collision/reaction cell (CRC) pressurised with oxygen as a reaction gas. SQ-KED is single quadrupole mode with CRC pressurised with helium as a collision gas and kinetic energy discrimination (KED) applied.

### 3.12. Extracellular Release of Inflammatory Cytokines

To measure the extracellular release of cytokines involved in the inflammatory process, THP-1 cells were plated in 12-well plates at 200,000 cells/well, and the differentiated macrophages were treated with 50 µg/mL of mineral fibres for 24 h. The IL-1β, MCP-1 and TNF-α content of the cell media was quantified by ELISA kit (Human IL-1beta ELISA Kit, TNF-alpha Human ELISA Kit and Human MCP1 SimpleStep ELISA**^®^** Kit, Abcam) following the manufacturer’s instructions. Data are the means ± SD of two independent experiments performed in duplicate.

### 3.13. RNA Extraction, cDNA Synthesis and qPCR Analyses

THP-1 monocytes were seeded in 6-well plates at 500,000 cells/well, and once differentiated, the macrophages were incubated with 50 µg/mL of mineral fibres for 6 h. The gene expression of inflammatory mediators, tumour necrosis factor-alpha (TNF-α a.n. NM_000594.4), interleukin-1β (IL-1β a.n. NM_000576.3), interleukin-6 (IL-6, NM_031168.2), interleukin-8 (IL-8, NM_000584.4) and monocyte chemoattractant protein-1 (MCP1, NM_002982), was then evaluated by qPCR relative to untreated cells. Total RNA was extracted using the NucleoSpin RNA, Mini kit (MACHEREY-NAGEL, Dueren, Germany) according to the manufacturer’s instructions. The quality and quantity of RNA were analysed using a NanoDrop spectrophotometer (Nanodrop Technologies, Wilmington, DE, USA). The cDNA was synthesised from 1 µg of RNA by using an iScript cDNA Synthesis Kit (Bio-Rad Laboratories, Milan, Italy). Each PCR reaction was performed in 10 µL containing: 4× master mix (biotechrabbit GmbH, Henningsdorf, Germany), 0.2 µM of each primer and 5 ng of cDNA. All samples were analysed in triplicate. The following thermal conditions were used: initial denaturation at 95 °C for three minutes, followed by 45 cycles with denaturation at 95 °C for 15 s, and annealing and elongation at 60 °C for 60 s. The fluorescence was measured at the end of each elongation step. Values were normalised to HPRT-1 (housekeeping gene) mRNA expression. All primers (Table 2) were designed using the Beacon Designer 7.0 software (Premier Biosoft International, Palo Alto, CA, USA) and obtained from TibMolBiol (Genova, Italy). Data analyses were obtained using the DNA Engine Opticon**^®®®^** 3 Real-Time Detection System Software program (3.03 version), and in order to calculate the relative gene expression compared to the undifferentiated (control) THP-1 monocyte sample, the comparative threshold Ct method was used [82] with the Gene Expression Analysis for iCycler iQ Real Time Detection System software (Bio-Rad, Milan, Italy). 

### 3.14. Statistical Analysis

Statistical analysis was performed using one-way ANOVA plus Tukey’s post-test (GraphPad Software, Inc., San Diego, CA, USA). Results with *p* values < 0.05 were considered significant.

## 4. Conclusions

The comparative study presented here highlights different mechanisms of toxicity as well as some similarities in the early responses of human M0, M1 and M2 macrophages towards three carcinogenic mineral fibres: crocidolite, chrysotile and erionite. The characteristics shown by these fibres are summarised in Table 3 and demonstrate that, although all three types of fibres are able to cause lung diseases, they act by different toxicity mechanisms owing to different rates of biodurability, the release of different toxic metal cargos and varying degrees of ROS and inflammatory mediator production through the activation of gene expression in human macrophages. 

All morphometric parameters (length and width) being equal, crocidolite seems to exert its pathological effects mostly due to its biodurability, with significant oxidative stress activation, DNA damage and the induction of gene expression of mediators involved in the maintenance of a chronic state of inflammation. Chrysotile also shows detrimental effects due to its low biodurability, which allows the rapid release of several toxic metal cargos both intra- and extracellularly, causing oxidative stress, DNA damage and the induction of gene expression of inflammatory mediators. On the other hand, biodurable erionite fibres exert adverse effects by other mechanisms because they induce decreased oxidative stress and decreased release of toxic metals. Conversely, a cation-exchange capacity able to alter intracellular cation concentrations is possible for this fibre, in contrast to CRO and CHR. Nevertheless, for erionite, significant DNA damage is also detected with the release of inflammatory cytokines, which is not accompanied by the upregulation of gene transcription in the first hours of stimulation.

It should be noted that the behaviours of the three macrophage phenotypes towards the mineral fibres surprisingly show several similarities, particularly in the production and release of pro-inflammatory mediators. It was observed that, when in contact with the investigated mineral fibres, the M2 macrophage phenotype, although known as a cell type that is recruited to counteract the detrimental effects of the inflammatory state of the tissue, apparently continues to support local inflammation by supplying a pro-inflammatory secretome.

Our comparative data on the three mineral fibres focus on the early stages of toxicity and macrophage activation and help to shed new light on the different complex mechanisms that lead to the onset of pulmonary pathologies due to mineral fibre inhalation. Furthermore, these data may help in the development of new prognostic tools for the ultimate classification of mineral particle and fibre toxicity. Indeed, a general quantitative model explaining the toxicity/carcinogenicity of mineral fibres is needed, both to resolve controversies on known mineral fibres with unclear classification and to help classify fibres with unknown toxicity. Our data provide a clearer picture of the chemical-physical fibre parameters contributing to the adverse effects on the physiology of humans and could be exploited for the development of a reliable fibre toxicity quantitative model. Additionally, further studies are needed, for instance, modelling more advanced states of inflammation upon fibre inhalation, possibly on 3D-reconstituted human lung tissues and organoids. These new human-relevant in vitro models will help to finally clarify the steps that lead to the long-lasting process of chronic pulmonary diseases and to produce new experimental tools for the classification of mineral fibres and particles, with a decrease in the costs and duration of testing relative to animal studies. 

## Figures and Tables

**Table 2:** List of primer pairs used for qPCR experiments in THP-1 human macrophages.

GENE	GenBank (a.n.)	Forward	Reverse	Size (bp)
IL-1β	NM_000576.3	TGATGGCTTATTACAGTGGCAATG	GTAGTGGTGGTCGGAGATTCG	140
IL-6	NM_001318095.2	CAGATTTGAGAGTAGTGAGGAAC	CGCAGAATGAGATGAGTTGTC	194
TNF-α	NM_000594.4	GTGAGGAGGACGAACATC	GAGCCAGAAGAGGTTGAG	113
IL-8	NM_000584.4	AATTCATTCTCTGTGGTATC	CCAGGAATCTTGTATTGC	127
MCP-1	NM_002982	CTTCTGTGCCTGCTGCTC	CTTGCTGCTGGTGATTCTTC	156
HPRT-1	NM_000194.3	GGTCAGGCAGTATAATCCAAAG	TTCATTATAGTCAAGGGCATATCC	144

**Table 3:** Summary of the chemical-biological effects of the three mineral fibres. Asterisks *, ** or ***, indicate a scale of increasing degree of an observed phenomenon.

	Crocidolite	Chrysotile	Erionite	Ref.
Biodurability	*** (66 yrs)	* (0.3 yrs)	*** (181 yrs)	[44]
Cell death	Mainly apoptosis	Apoptosis and cell lysis	Apoptosis and cell lysis	
ROS production	***	**	**	
Redox-active metal release	Fe	Mg, Fe, Ni, Cr, Co	Al	
DNA damage	***	***	***	
Cytokine release	***	***	***	
Inflammatory gene upregulation	***	**		
Cation-exchange capacity (CEC)			***	[9]

## Data Availability

The present article reports only original data that have not been published elsewhere.

## Supplementary Materials

The following supporting information can be downloaded at: https://www.mdpi.com/article/10.3390/ijms23052840/s1.
