# Supplementary material for: The Acute Toxicity of Mineral Fibres: A Systematic In Vitro Study Using Different THP-1 Macrophage Phenotypes
Source: Int J Mol Sci. 2022 Mar 4;23(5):2840. doi: 10.3390/ijms23052840 (PMC8911508; doi:10.3390/ijms23052840)
Supplement: Supplementary file 1 [file ijms-23-02840-s001.zip › ijms-1614845-supplementary.pdf]

International Journal of *Molecular Sciences*

MDPI

Article

The Acute Toxicity of Mineral Fibres: A Systematic In Vitro Study Using Different THP-1 Macrophage Phenotypes

Serena Mirata [1,2] Vanessa Almonti [2,3], Dario Di Giuseppe [4], Laura Fornasini [5], Simona Raneri [5], Stefania Vernazza [2,3], Danilo Bersani [6], Alessandro F. Gualtieri [4], Anna Maria Bassi [2,3] and Sonia Scarfì [1,2,*]

[1] Department Earth, Environment and Life Sciences, University of Genova, 16132 Genova, Italy; serenamira94@gmail.com
[2] Inter-University Center for the Promotion of the 3Rs Principles in Teaching & Research (Centro 3R), 56122 Pisa, Italy
[3] Department Experimental Medicine, University of Genova, 16132 Genova, Italy; vanessaalmonti@gmail.com (V.A.); stefania.vernazza@yahoo.it (S.V.); anna.maria.bassi@unige.it (A.M.B.)
[4] Department of Chemical and Geological Sciences, University of Modena and Reggio Emilia, Via G. Campi 103, I-41125 Modena, Italy; dario.digiuseppe@unimore.it (D.D.G.); alessandro.gualtieri@unimore.it (A.F.G.)
[5] ICCOM-CNR—Institute of Chemistry of OrganoMetallic Compounds, National Research Council, Via G. Moruzzi 1, 56124 Pisa, Italy; laura.fornasini@pi.iccom.cnr.it (L.F.); simona.raneri@pi.iccom.cnr.it (S.R.)
[6] Department of Mathematical, Physical and Computer Sciences, University of Parma, Parco Area delle Scienze 7/A, 43124 Parma, Italy; danilo.bersani@unipr.it
* Correspondence: soniascarfi@unige.it; Tel.: 0039-010-335-0227

Supplementary materials and methods:

The following mineral fibres have been selected for the study:

1) UICC standard crocidolite (South African, NB #4173-111-3) with chemical formula $(Na_{1.96}Ca_{0.03}K_{0.01})_2(Fe^{2+}_{2.34}Fe^{3+}_{2.05}Mg_{0.52})_{4.91}(Si_{7.84}Al_{0.02})_{7.86}O_{21.4}(OH)_{2.64}$ [48]. Sample contain minor (<1 wt%) impurities of hematite, magnetite, and quartz. This mineral fibre has been widely used as a positive control in numerous in vitro and in vivo studies [49, 80-82]. Full characterisation of the sample is available in the following works: Gualtieri et al. [48] and Pacella et al. [36].

2) Chrysotile from Balangero (Torino, Italy) with chemical formula $(Mg_{5.81}Fe^{2+}_{0.15}Al_{0.27}Fe^{3+}_{0.09}Cr_{0.01})_{6.33}Si_{3.97}O_{10}(OH)_{7.11}$ [32]. Sample contain minor impurities of antigorite, balangeroite, calcite, clinochlore, diopside, dolomite, magnetite, microcline, plagioclase, talc. Complete characterisation of the sample is available in Pollastri et al. [32] and Gualtieri et al. [48]. The cyto-genotoxicity of chrysotile from Balangero has recently been studied in the work of Gualtieri et al. [14].

3) Fibrous erionite-Na from Jersey (Nevada USA) $[(Na_{5.35}K_{2.19}Ca_{0.15}Mg_{0.11}Ti_{0.05})_{7.85}(Si_{28.01}Al_{7.90})_{35.91}O_{72\cdot 28}\cdot 13H_2O]$ with traces of clinoptilolite [28]. Complete characterisation of the sample is available in Pollastri et al. [32] and Gualtieri et al. [28].

Supplementary Figures

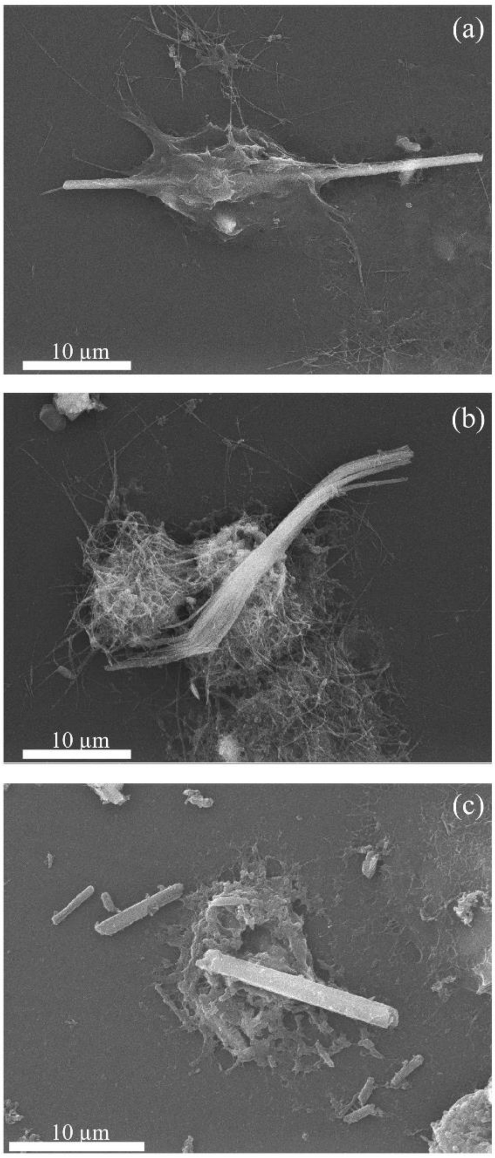

Figure S1. High-resolution FEG-SEM microphotographs of (**a**) M0-THP-1 cell interacting with a crocidolite fibre, (**b**) M0-THP-1 cell interacting with a chrysotile fibre, (**c**) M0-THP-1 cell interacting with an erionite fibre.
